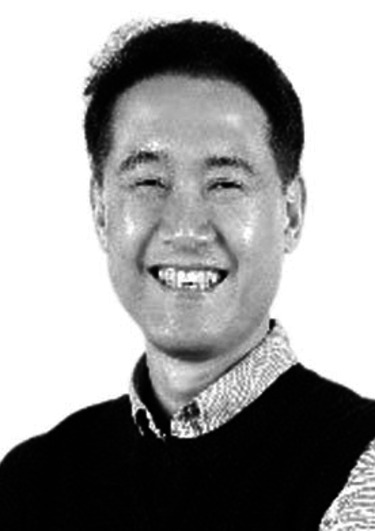# Indoor Organic Photovoltaics for Self‐Sustaining IoT Devices: Progress, Challenges and Practicalization

**DOI:** 10.1002/cssc.202100981

**Published:** 2021-06-17

**Authors:** Muhammad Jahandar, Soyeon Kim, Dong Chan Lim

**Affiliations:** ^1^ Energy and Electronic Materials Center Korea Institute of Materials Science (KIMS), Korea Changwon 51508 Republic of Korea

**Keywords:** energy storage, Internet of Things, organic materials, photovoltaics, solar cells

## Abstract

Indoor photovoltaics (IPVs) have great potential to provide a self‐sustaining power source for Internet‐of‐Things (IoT) devices. The rapid growth in demand for low‐power IoT devices for indoor application not only boosts the development of high‐performance IPVs, but also promotes the electronics and semiconductor industry for the design and development of ultra‐low‐power IoT systems. In this Review, the recent progress in IPV technologies, design rules, market trends, and future prospects for highly efficient indoor photovoltaics are discussed. Special attention is given to the progress and development of organic photovoltaics (OPVs), which demonstrate great possibilities for IPVs, owing to their bandgap tunability, high absorbance coefficient, semitransparency, solution processability, and easy large‐area manufacturing on flexible substrates. Highly efficient indoor organic photovoltaics (IOPVs) can be realized through designing efficient donor and acceptor absorber materials that have good spectral responses in the visible region and better energy‐aligned interfacial layers, and through modulation of optical properties. Interfacial engineering, photovoltage losses, device stability, and large‐area organic photovoltaic modules are surveyed to understand the mechanisms of efficient power conversion and challenges for IOPVs under indoor conditions as a self‐sustaining power source for IoT devices. Finally, the prospects for further improve in IOPV device performance and practical aspects of integrating IOPVs in low‐power IoT devices are discussed.

## Graphical abstract

**Table of Contents**: Organic photovoltaics demonstrate great potential for indoor photovoltaic applications, owing to their bandgap tunability, high absorbance coefficient, semitransparency, solution processability, and easy large‐area manufacturing on flexible substrates. Organic photovoltaics can provide a self‐suitable power source for Internet‐of‐Things (IoT) systems under indoor or low light intensity conditions.

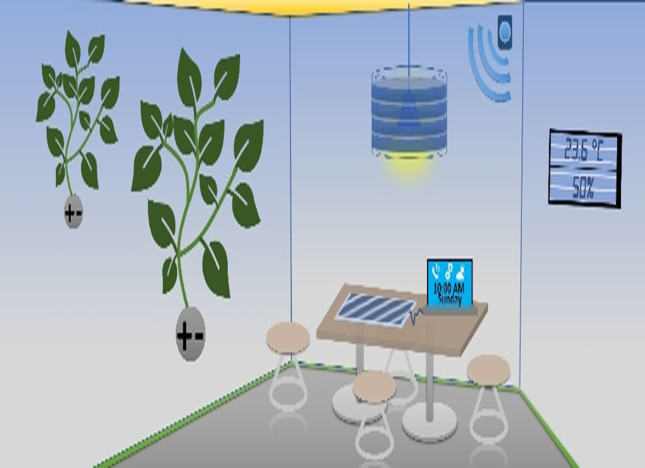

## 1 Introduction

In the past few years, the rapid development of low power internet of things (IoT) devices that are embedded with different type of sensors, software and other supportive technologies for the purpose of communicating and exchanging data with their internal states or external environment including other electronic devices and systems through wireless network or internet, ascends the demand of self‐sustaining power sources.[^1^, ^2^, ^3^] To date, most of the low power IoT devices installed in homes, offices, markets or factories are powered by disposable or rechargeable batteries. In coming years, the number of such IoT devices are expected to grow in billions. The limited lifetime and a huge number of battery replacement can cause serious maintenance and environment issues. An enormous power supply will be required to overcome the needs of such a vast IoT system. Among renewable energy harvesting sources, photovoltaic technologies have shown great potential to efficiently convert the indoor ambient energy into electricity with better reliability and operational lifetimes. They can be easily integrated with low power IoT devices as sole or supporting the battery to extend its lifetime.[^4^, ^5^, ^6^, ^7^, ^8^]

The enormous development and promising future of photovoltaic technologies makes them prominent contender among other renewable energy sources. Among these photovoltaic technologies, crystalline silicon (c‐Si) and amorphous silicon (a‐Si) photovoltaics still have the major market share for outdoor applications with excellent large area power conversion efficiencies (PCEs) and stability.[^9^, ^10^] However, other emerging photovoltaic technologies, such as perovskite solar cells (PSCs), dye sensitized solar cells (DSSCs) and organic photovoltaics (OPVs) have undergone rapid development, owing to excellent properties such as bandgap tunability, easy solution processability, transparency, low cost, light weight, and mechanical flexibility. In terms of their device performance under standard conditions (1 sun illumination, 100 mW cm^−2^), PSCs are approaching the PCE of c‐Si solar cells with record efficiency of 23.3 %, whereas OPVs reported a record PCE of 18.22 %.[^11^, ^12^] Similarly, DSSCs shown a record PCE of 14.3 %.^[13]^ Despite the higher efficiencies of these next‐generation photovoltaics, device stability is the major challenge that need to be addressed. Although, at the present these emerging photovoltaics technologies cannot compete the conventional inorganic photovoltaics in terms of PCEs and stability under standard illumination conditions but due to their unique properties they are undoubtedly more preferable for the applications such as portable electronics, wearable electronics and IoT systems to provide sustainable power sources in low light or indoor environments. Furthermore, relatively favorable indoor conditions compare to the harsh outdoor environment conditions such as heat, solar irradiations and fluctuating weather can provide a pathway for prolonged device stability and performance.[^14^, ^15^, ^16^, ^17^, ^18^]

Interestingly, table turns when we compare the device performance of inorganic photovoltaics with next‐generation solution processed organic or hybrid photovoltaics under ambient light sources. The requirements of high‐performance photovoltaics under ambient light sources are different from those under standard 1 sun illumination, as the emission spectra of modern indoor lighting sources such as fluorescent lamps (FL) and light‐emitting diodes (LEDs) are much narrower (400 to 750 nm) than the standard solar spectrum, which covers ultraviolet, visible, and infrared regions. Therefore, the device optimization strategies adopted to realize high‐performance photovoltaics under standard 1 sun illuminations could alter under indoor light illuminations.[^5^, ^19^] The mismatch between absorbance spectra of photo absorber materials and emission spectra of light sources could lead to the photovoltage losses (*V*_loss_). On the other hand, considerably low light intensities of indoor light sources (25–300 μW cm^−2^, 100—1000 lux) compared to standard 1 sun illuminations (AM 1.5 G, 100 mW cm^−2^) leads to low charge carrier densities in indoor photovoltaics (IPVs) which could promote Shockley‐Read‐Hall recombination resulting from trap states.[^1^, ^20^, ^21^] Therefore, careful considerations in choice of photoactive materials, device architecture, and interfacial engineering are required to optimize high‐performance indoor photovoltaics.

In this Review, we generally discuss the recent advances in IPVs technologies, design rules, market trends and future prospect for highly efficient IPVs. Furthermore, a prime focus is given to the research and investigation of indoor organic photovoltaics (IOPVs) which demonstrate great possibilities for indoor photovoltaics due to their extraordinary properties of bandgap tunability, high absorbance coefficient, semitransparent, solution processability and easy large area manufacturing on flexible substrates.[^12^, ^22^, ^23^, ^24^, ^25^, ^26^, ^27^, ^28^] The ultimate IOPVs can be realized through designing efficient donor and acceptor absorber materials, better energy aligned interfacial layers and through modulation of optical properties. As the indoor light sources much differ in characteristics of spectrum wavelength and intensities compared to standard 1 sun illumination. A detailed discussion about the IPVs device optimization to realize for indoor applications will be presented. Interfacial engineering, photovoltage losses, device stability and large area OPV modules are surveyed exclusively to understand the mechanism of efficient power conversion and challenges for IPVs under indoor environment as a self‐sustaining power source for IoT devices. Finally, we will discuss the prospect to further improve the IPVs device performance and practicalization of IPVs integrated low power IoT devices.

## 2 Recent Progress in Indoor Photovoltaic Technologies

**Figure 1:**
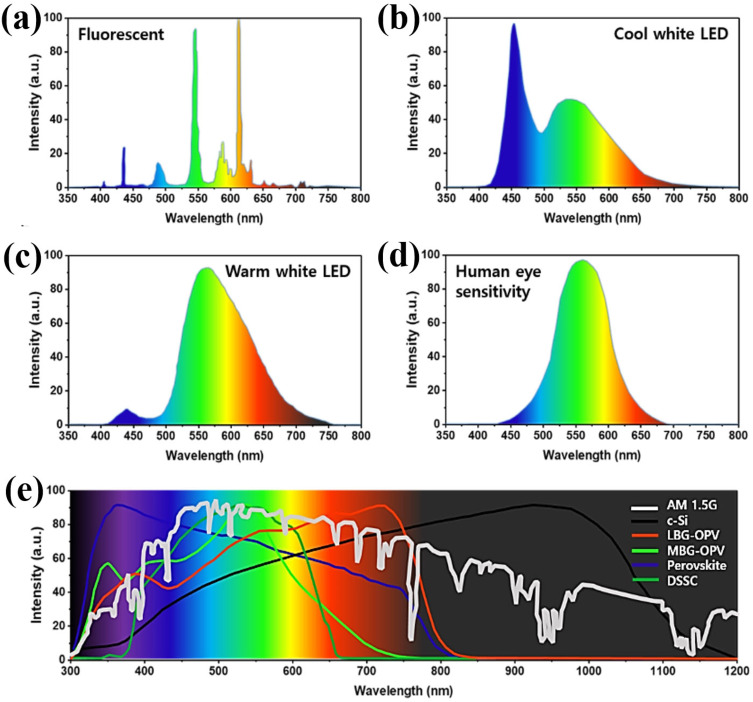
Emission spectra of indoor light sources: (a) Fluorescent lamp; (b) cool white LED; (c) warm white LED. (d) Human eye sensitivity spectrum. (e) standard solar spectrum (AM 1.5G) overlaid with spectral response of various photovoltaic absorber materials. Reproduced with permission from ref. [5]; copyright 2019, Bentham Science Publishers.

These days most commonly used artificial indoor light sources are fluorescent or LED lamps that are highly economical, long life‐time and energy efficient. The basic properties of these light sources can be different in terms of color temperature, illuminations intensities and radiation wavelength range according to the environment and application requirements. In general, the indoor illumination intensities for different type of work spaces such as public areas, warehouses, homes, schools, offices, supermarkets and electronic workshops are approximately 50, 150, 200, 300, 500, 750 and 1500 lux respectively.[^14^, ^29^] Although these illumination intensities are much lower (ca. 1000 times) than solar light or standard simulated solar light intensities, they are sufficient to supply mW power to 10×10 cm^2^ scale IPVs. Particularly, the indoor light sources have significantly different spectral range, composition and illumination intensities compared to the sunlight and standard simulator illuminations. The emission spectra of FL and LED lights (Figure 1a–c) are quite narrow compared to standard 1 sun (AM 1.5 G) solar spectrum (Figure 1e). The spectral sensitivity of the indoor photovoltaics must overlap completely with that of the human eye (Figure 1d) for better performance under all indoor light sources provided for human use including FL and LED lamp light sources.^[5]^

**Figure 2:**
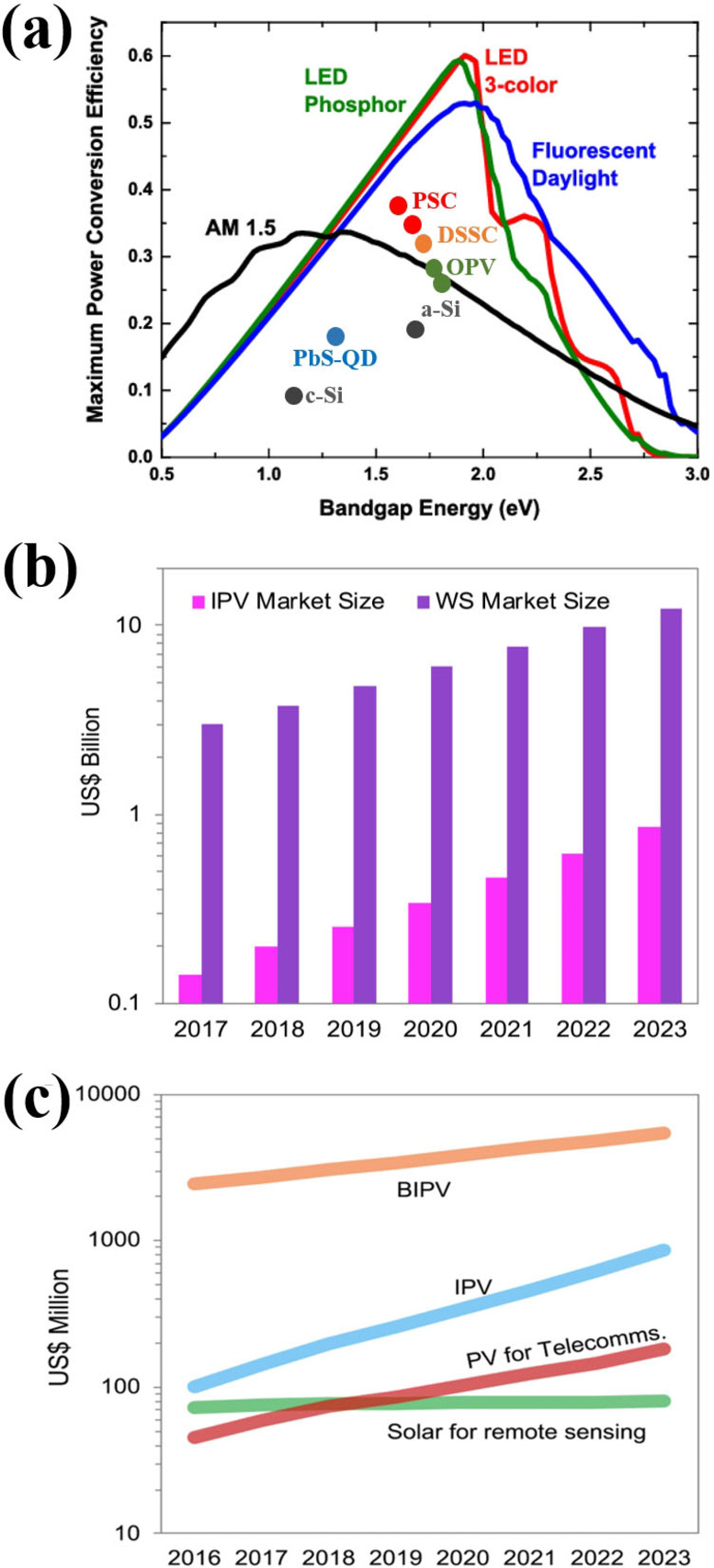
(a) Calculated maximum PCE versus material bandgap under various lighting for indoor conditions. Reproduced with permission from ref. [30]; copyright 2015, IEEE. (b) Projected size of the indoor photovoltaics market and of the wireless sensors in billions of dollars and (c) expected capacity trends of alternative markets for photovoltaic technologies in the coming years. Reproduced with permission from ref. [1]; copyright 2019, Cell Press.

In general, photovoltaic device performance is evaluated under standard illumination conditions (AM 1.5G, 100 mW cm^−2^). The fundamental difference between indoor and outdoor applications is the spectrum and intensity of the light source. Figure 1e shows the spectral response of various photovoltaic technologies.^[30]^ It can be seen clearly that the c‐Si have good spectral matching with standard solar spectrum (AM 1.5G), whereas the next‐generation photovoltaic technologies (OPVs, PSCs, and DSSC) have good spectral response in the visible region. Figure 2a shows the comparison of different light sources (phosphor LED, 3‐color LED, fluorescent day light and AM 1.5), where the efficiencies are calculated by the spectral range of the light sources and not their intensities. The maximum achievable efficiency of the photovoltaic device under outdoor or indoor environment entirely depends on the device type and bandgap of the photoactive layer. A low bandgap photoactive layer device can produce maximum power conversion efficiency under outdoor solar light or standard illumination conditions, whereas a higher bandgap photoactive layer device could harvest maximum efficiency under indoor illumination conditions. The best experimentally obtained PCEs of different IPV devices with respect to the bandgap of their light absorber layers are shown in Figure 2a.[^2^, ^31^, ^32^, ^33^, ^34^, ^35^, ^36^] These PCEs of IPVs are still lower than that of theoretical PCEs (51–57 %) with optimal energy bandgap between 1.82 and 1.96 eV.^[37]^ Therefore, considering the theoretical PCEs limits of IPVs, there is still a lot of performance improvement required. There are few fundamental factors that need to be considered carefully to obtain an excellent photovoltaic device performance under indoor light conditions. Firstly, the IPVs should have best photo‐response spectrum that matches the indoor light spectrum. The absorbance spectra of IPVs photoactive layer should overlap with emission spectra of indoor light source. Secondly, the external quantum efficiency (EQE) of the IPVs should be high to realize the maximum conversion of incident light photons into current as well as to subside the thermalization of photogenerated charges. Thirdly, the trap assisted charge recombination should be reduced, as low light intensities cause low carrier densities that can promote the trap assisted recombination mechanism. Lastly, the energy losses (*E*_loss_ = *E*_g_−*qV*_OC_, where *E*_g_ is the band gap, *q* is the elementary charge and *V*_OC_ is the open‐circuit voltage) of the IPVs should be low to realize the maximum open‐circuit voltage (*V*_OC_), as reduced energy losses and high *V*_OC_ under low light intensities could boost the IPVs device performance significantly.[^2^, ^5^, ^38^]

To date, the record efficiency of c‐Si photovoltaic cells under standard illumination conditions is 27.6 %, which is much higher than competitor photovoltaic technologies such as PSCs, OPVs and DSSCs. However, the situation completely changes under indoor light illuminations. Reich et al. demonstrated a comprehensive study to understand the effect of indoor light illuminations on device parameters of mono‐ and multi‐crystalline silicon (c‐ and mc‐Si) and hydrogenated amorphous silicon (a‐Si : H) photovoltaic cells and compared the device performance under standard 1 sun illuminations and artificial light conditions (white LED light, 1000 lux).^[35]^ Under standard illuminations, c‐Si and mc‐Si shown the PCEs of 18.2 and 16.8 %, which drop significantly to 5.6 and 3.7 % under LED light sources, respectively. In contrast, a‐Si : H exhibited a PCE of 7.7 % under standard illuminations, which increased to 19–21 % under LED light source. The poor spectral response of c‐Si and mc‐Si to LED emission spectrum cause serious drop in *V*_OC_ and fill factor (FF) resulting in poor device performance under indoor light source. Particularly, the absorbance spectra from 750 to 1100 nm are redundant and worse, lead to the photovoltage losses. Similarly, Christie et al. compared the device performance of copper indium gallium selenide (CIGS) photovoltaics cell having absorbance spectrum wavelength from 400 to 1100 nm under standard AM 1.5G solar simulator lamp and LED lamp, and observed a drop in PCE from 2.64 % to 2.01 %.^[29]^ Hou et al. analyzed the device performance of lead sulfide (PbS) quantum dots (QDs) based photovoltaic cells having bandgap of 1.24 eV and surprisingly obtained improved device performance under FL light (PCE: 18.1 % at 1000 lux) compared to standard 1 sun illumination (PCE: 9.55 %) due to higher EQE in 350 to 800 nm region.^[32]^ Interestingly, several previous studies demonstrated that the PSCs, OPVs and DSSCs can convert indoor lights into electricity more efficiently than other inorganic technologies because of their highly tunable optical absorption, large absorption coefficient, and small leakage currents under low light environment.[^7^, ^39^, ^40^, ^41^, ^42^, ^43^, ^44^, ^45^] Recently, Noh et al. reported a record PSCs efficiency of 37.2 % under indoor illumination conditions (1000 lux, 6500 K LED) with excellent ambient device stability of more than 800 h.^[33]^ Similarly, Harrison et al. demonstrated a PCE of 28.1 % for fullerene based OPVs under fluorescent lamp of 1000 lux and Ma et al. reported record efficiencies of 28.11 % (290 lux) and 30.89 % (1650 lux) under LED illuminations (3000 K) for nonfullerene acceptor based OPVs.[^34^, ^46^] On the other hand, Cao et al. reported a record PCE of 32 % for DSSCs under fluorescent lamp at 1000 lux.^[36]^ All these indoor high efficiencies of next‐generation photovoltaics are the result of excellent photo spectral response in the visible region and reduced energy losses with better energy alignment management.

Indoor photovoltaics have great potential to provide self‐sustaining power sources for IoT devices. The rapid growth in the demand of low power IoT devices for indoor applications not only boost the development of high‐performance IPVs, also promote the electronic and semiconductor industry for the design and development of ultra‐low power IoT systems. According to global market, technologies and devices for energy harvesting reports, in 2017, global IPV market was worth $140 million which was insignificant compared to solar power modules, which was over $100 billion. With the growth of IoT systems market, the forecast of the IPVs market are to reach $850 million by 2023 (Figure 2b). This signifies a compound annual growth of 70 % compared to the present market and makes the IPVs technologies fastest growing market among other non‐traditional photovoltaic markets (Figure 2c).[^1^, ^4^] Furthermore, the strategies to reduce the production cost and device performance improvement can further boost the IPVs market.

## 3 Progress of Indoor Organic Photovoltaics

Over the last three decades, organic photovoltaics have attained tremendous progress through the development of organic photovoltaic materials and device engineering. The photo absorber layer of OPVs consist of donor and acceptor materials which are blended together to form a bulk heterojunction (BHJ). This BHJ is represented as active layer, absorbs light photons and converts them into free charge carriers in OPVs. The development of organic light absorber materials has played an important role in realizing the PCEs for OPVs from 1 % to the state of the art PCEs over 18 %.[^12^, ^47^, ^48^, ^49^, ^50^, ^51^] The first major breakthroughs in OPV technology was the adoption of C_60_ fullerene and its derivatives such as [6,6]‐phenyl‐C_61_‐butyric acid methyl ester (PCBM).^[52]^ Owing to high electronegativity and electron mobility, PCBM have been widely used as electron acceptor and become a standard acceptor material in OPV devices. The enormous efforts have been devoted to design and develop polymer or small molecular donor materials to ensure a good compatibility with PCBM acceptor materials in terms of the absorbance spectra and molecular energy levels alignment. The prime focus of the researchers was to develop low bandgap donor materials to cover the maximum solar spectrum for high efficiency OPVs. However, the limited control of tunability of optoelectronic properties and large energy losses of fullerene‐based acceptors can only translate a maximum PCEs around 12 %.^[53]^ A new era of OPVs started when researchers developed efficient nonfullerene acceptors (NFAs). An innovative nonfullerene acceptor molecule ITIC with PBDB−T polymer donor set a new landmark for OPVs with a remarkable improvement in PCEs.^[56]^ Since then, exclusive researches have been conducted to tune the optical, electronic, and crystalline properties of NFAs to obtain highly efficient OPVs. Most recently, a new class of NFAs Y6 and its derivatives have demonstrated remarkable PCEs of 15 to 18 % with suitable donor materials due to broad absorption spectra and reduced energy losses.[^12^, ^55^, ^56^]

Previously, most of the efforts were devoted for the development of low bandgap donor materials for outdoor light harvesting and not many attentions were given for indoor light harvesting. One basic limiting factor that hinder the device performance of OPVs under low‐light illuminations was the higher energy losses of fullerene‐based acceptors. Although many medium‐ to high‐bandgap donor materials incorporating with PCBM to fabricate BHJ have been reported, the majority of them have *V*_OC_ values less than 0.80 V under standard illumination, which further reduce to lower *V*_OC_ through extra energy losses under low‐illumination conditions.[^14^, ^38^, ^57^, ^58^, ^59^] However, the emergence of NFAs that have demonstrated low energy losses with suitable donor materials has broadened the choice of acceptor materials for both indoor and outdoor photovoltaic applications. To develop high‐performance IPVs, the choice of photoactive materials that have good spectral response with indoor lighting environment and energy level alignment is the most important factor in minimizing energy losses for the realization of high *V*_OC_ values. Furthermore, the selection of efficient charge transporting layers, interfacial engineering, and optical management can also play an important role in reducing energy losses, carrier recombination and leakage currents to improve the current density under low light illumination conditions.

### 3.1 Indoor organic photovoltaic materials

**Figure 3:**
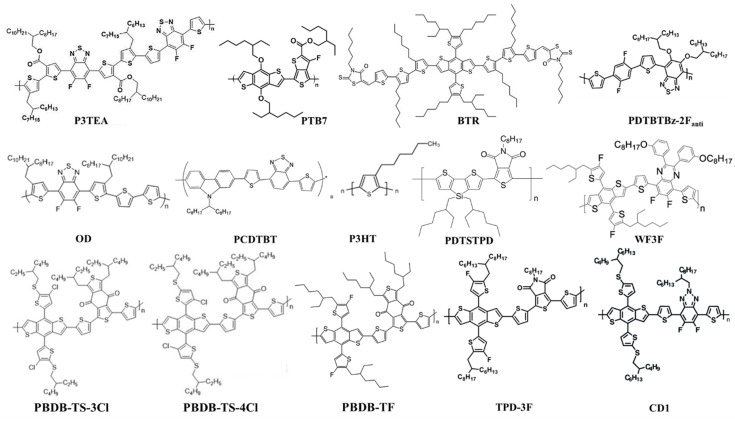
Chemical structures of donor materials applied in high‐performance indoor organic photovoltaics.

**Figure 4:**
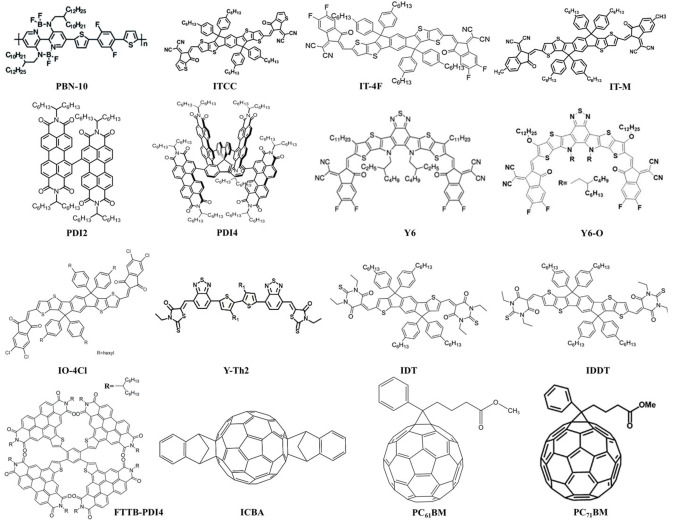
Chemical structures of acceptor materials applied in high‐performance indoor organic photovoltaics.

The development of OPVs is the outcome of continuous innovations in materials science. Nowadays, photoactive materials for OPVs can be classified in five major categories such as polymer donor, polymer acceptor, fullerene acceptor, small molecule donor and small molecule acceptor. Figures 3 and 4 show the chemical structures of some donor and acceptor materials utilized in high‐performance IOPVs. In last few years, medium bandgap polymer/small molecule donors with fullerene‐based acceptors reported IOPVs having PCEs of 8–28 % under indoor light illumination conditions.[^14^, ^17^, ^19^, ^60^, ^61^, ^62^] Yang et al. investigated the P3HT polymer with different fullerene derivatives (PC_60_BM and ICBA) and obtained PCEs of 8.90 and 13.05 % for P3HT:PC_60_PB and P3HT:ICBA photoactive layers respectively, under 500 lux LED lamp.^[60]^ The high performance of P3HT with ICBA compared to PC_60_BM acceptor material is due to better energy alignment. The higher energy offsets between the highest occupied molecular orbital (HOMO) of P3HT donor and lowest unoccupied molecular orbital (LUMO) of ICBA acceptor results in higher *V*_OC_ compared to PC_60_BM acceptor, resulting in higher PCEs. Similarly, another excellent donor polymer PBDTTT‐EFT (PCE10) that have relatively broader absorption range compare to P3HT can produce PCE of 13.20 % under 500 lux LED lamp with blend of PC_70_BM acceptor.^[60]^ Although, PCE10:PC_70_BM BHJ can have higher *J*_SC_ compared to P3HT:ICBA BHJ but relatively lower *V*_OC_ and FF, resulting in comparable indoor device performance (Table 1). Harrison et al. demonstrated IOPVs with PCDTBT and PTB7 donor polymers blend with fullerene‐based acceptor materials for comparison under low‐light illuminations using fluorescent lamp.^[14]^ PCDTBT based devices were found to be the one of the best performing system, generating higher *V*_OC_ (0.72) corresponding to 16.6 % PCEs at 300 lux. Although, PTB7 based devices showed the higher efficiency compared to PCDTBT based device under standard 1 sun illuminations but due to lower *V*_OC_ (0.61 V) under low‐light illuminations can only achieve PCE of 14.6 %. Ranbir et al. design and synthesis a series of donor polymer materials namely WF3, WF3S and WF3F. Among these donor materials, WF3F can effectively minimize the bimolecular recombination with reduced series resistance, increased shunt resistance and better film morphology. As a result, WF3F:PC_71_BM BHJ can exhibit a PCE of 17.34 % with *V*_OC_: 0.69 V under 500 lux LED source.^[62]^ Moreover, Shim et al. demonstrated PDTBTBz‐2F_anti_:PC_71_BM OPVs which exhibits an excellent spectrum matching with indoor lighting environment, resulting in an excellent power absorption ratio.^[63]^ These optical properties contribute to performance of the PDTBTBz‐2F_anti_:PC_71_BM based IOPVs with a high PCE of 23.1 % having *V*_OC_: 0.81 V under 1000 lux LED illuminations. Another class of OPV is based on small‐molecule donor materials and fullerene‐based acceptors. A small‐molecule donor BTR, which is based on a benzodithiophene (BDT) core and a rhodamine end group connected by terthiophene units, where BDT core has a thiophene side chain with attached 2‐ethylhexyl and hexyl groups.^[17]^ This distinct arrangement of alkyl chains permits interesting morphological properties. Furthermore, BTR small‐molecule donor with bandgap of 1.80 eV, high EQE and *V*_OC_ is suitable for efficient indoor light harvesting. A PCE of 28.1 % was reported for BTR:PC_71_BM OPVs under indoor illumination conditions, which is the highest PCE for IOPVs with fullerene‐based acceptor materials.

**Table 1:** Summary of photovoltaic device properties of previously reported indoor organic photovoltaics.

Light source	Active layer	HTL	ETL	*V*_OC_ [V]	*J*_SC_ [μA cm^−2^]	FF [%]	PCE [%]	Ref.
Fullerene acceptor‐based indoor organic photovoltaics								
LED (500 lux)	P3HT:PC_60_BM	PEDOT:PSS	Ca	0.43	62.0	59.0	8.90	[60]
LED (500 lux)	P3HT:ICBA	PEDOT:PSS	Ca	0.73	50.0	63.0	13.05	[60]
LED (500 lux)	PTB7‐Th:PC_70_BM	PEDOT:PSS	Ca	0.59	66.0	58.0	13.20	[60]
FL (300 lux)	PCDTBT:PC_71_BM	PEDOT:PSS	Ca	0.72	27.7	69.3	16.60	[14]
FL (300 lux)	PTB7:PC_71_BM	PEDOT:PSS	Ca	0.61	28.6	69.5	14.60	[14]
LED (500 lux)	WF3F:PC_71_BM	ZnO	MoO_3_	0.69	63.5	67.4	17.34	[62]
LED (1000 lux)	PDTBTz‐2F_anti_:PC_71_BM	PEDOT:PSS	Ca	0.81	112.4	70.4	23.1	[63]
FL (1000 lux)	BTR:PC_71_BM	PEDOT:PSS	Ca	0.79	133	75.0	28.1	[17]

Nonfullerene acceptor‐based indoor organic photovoltaics								
FL (500 lux)	PBDB‐TS:IT‐4F	MoO_3_	ZnO	0.36	66.8	30.9	5.3	[64]
FL (500 lux)	PBDB‐TS‐3Cl:IT‐4F	MoO_3_	ZnO	0.64	62.8	72.2	20.4	[64]
FL (500 lux)	PBDB‐TS‐4Cl:IT‐4F	MoO_3_	ZnO	0.64	64.9	73.9	21.7	[64]
LED (500 lux)	PBDB‐TF:IT‐M	PEDOT:PSS	PFN‐Br	0.88	55.4	75.4	22.8	[65]
LED (1000 lux)	PBDB‐TF:IT‐4F	PEDOT:PSS	PFN‐Br	0.71	113.0	78.0	20.8	[19]
LED (1000 lux)	PBDB‐TF:ITCC	PEDOT:PSS	PFN‐Br	0.96	95.8	72.0	22.0	[19]
LED (200 lux)	PBDB‐TF:IO‐4Cl	PEDOT:PSS	PFN‐Br	1.03	18.2	71.5	22.2	[2]
LED (500 lux)	PBDB‐TF:IO‐4Cl	PEDOT:PSS	PFN‐Br	1.07	45.1	76.8	24.6	[2]
LED (1000 lux)	PBDB‐TF:IO‐4Cl	PEDOT:PSS	PFN‐Br	1.10	90.6	79.1	26.1	[2]
LED (1000 lux)	CD1:PBN‐10	PEDOT:PSS	Ca	1.14	105.0	65.4	21.7	[15]
FL (1000 lux)	CD1:PBN‐10	PEDOT:PSS	Ca	1.14	120.0	66.2	26.2	[15]
LED (1000 lux)	CD1:ITIC	PEDOT:PSS	Ca	0.77	107.0	67.5	15.4	[15]
FL (1000 lux)	CD1:ITIC	PEDOT:PSS	Ca	0.78	116.0	68.1	17.9	[15]
LED (1010 lux)	P3TEA:FTTB‐PDI4	PEDOT:PSS	PDI‐NO	1.00	110.8	66.6	23.9	[46]
LED (1650 lux)	P3TEA:FTTB‐PDI4	PEDOT:PSS	PDI‐NO	1.01	179.7	66.8	24.2	[46]
LED (1650 lux)	PBDB‐TF:Y6‐O	PEDOT:PSS	PFN	0.83	239	57.0	22.4	[46]
LED (1650 lux)	PBDB‐TF:Y6‐O	PEDOT:PSS	PDI‐NO	0.84	245	76.0	30.8	[46]

Ternary indoor organic photovoltaics								
LED (300 lux)	PCDTPT:PDTSTPD:PC_71_BM	PEDOT:PSS	LiF	0.73	33.1	61.1	18.9	[69]
FL (300 lux)	PCDTPT:PDTSTPD:PC_71_BM	PEDOT:PSS	LiF	0.73	33.3	63.5	20.8	[69]
LED (1000 lux)	OD:PC_71_BM:IDT	V_2_O_5_	ZnO	0.63	132.9	71.7	21.1	[70]
LED (1000 lux)	OD:PC_71_BM:IDDT	V_2_O_5_	ZnO	0.64	126.3	64.2	19.0	[70]
LED (1000 lux)	OD:PC_71_BM:PDI2	V_2_O_5_	ZnO	0.64	127.1	70.0	20.5	[70]
LED (1000 lux)	OD:PC_71_BM:PDI4	V_2_O_5_	ZnO	0.64	119.2	67.8	18.2	[70]
LED (1000 lux)	PBDB‐TF:Y−Th2:Y6	PEDOT:PSS	PDI‐NO	0.70	32.0	74.0	22.7	[71]

Although, the fullerene‐based acceptor materials have good compatibility with LED and FL sources emission spectra due to absorption in UV‐visible region but relatively deeper LUMO energy level cause larger energy losses resulting in low *V*_OC_ and weak absorbance in visible region leading to low current densities. In addition, the difficult bandgap tunability of fullerene derivative hinders the further improvement of optoelectronic properties. Consequently, nonfullerene acceptors materials including polymer and small molecule acceptor materials explored due to their easy synthesis and bandgap tunability. Kwon et al. synthesized a series of copolymers based on PBDB‐TS and investigated the effects of chlorine substitution of its thiophene‐substituted benzodithiophene (BDT−Th) unit.^[64]^ The polymer donors PBDB‐TS, PBDB‐TS‐3Cl and PBDB‐TS‐4Cl were blend with IT‐4F NFA to fabricate photo absorber layer. The chlorination of the polymer resulted in efficient charge transport, suppressed leakage current and an increased *V*_OC_ of the IOPVs resulted in PCE from 5.3 % for PBDB‐TS to 21.7 % for PBDB‐TS‐4Cl under 500 lux fluorescence illuminance. Hou et al. studied the PBDB‐TF donor and IT−M NFA for IOPVs to investigated the effect of the introduced external resistance and active layer thickness on device performance of the IOPVs and obtained PCE of 22.8 % under 500 lux LED lamp.^[65]^ They also fabricated the PBDB‐TF:ITCC, PBDB‐TF:IT‐4F and the PBDB‐TF:PC_71_BM OPV devices to verify the possibility of large‐area processability.^[19]^ Under 1000 lux LED illuminations, ITCC, IT‐4F and PC_71_BM based IOPV achieved 0.96, 0.71 and 0.78 V under illuminance of 1Sun and 1000 lux LED, respectively, compared with PC_71_BM based device of 0.945 V and 0.784 V, respectively. The ITCC based IOPV devices PCE (22.0 %) exceeded that of PC_71_BM (18.1 %) and IT‐4F (20.8 %) based IOPVs under 1000 lux LED illuminations due to better spectral matching and low energy losses. Further, they also synthesized a new wide bandgap NFA IO‐4Cl blended with PBDB‐TF which achieved a PCE over 26 % and a large *V*_OC_ of 1.1 V under 1000 lux of LED light illumination.^[2]^ PBDB‐TF:IO‐4Cl IOPV device had outstanding stability. Liu et al. reported a medium bandgap donor polymer namely CD1 with PBN‐10 NFA to construct all‐polymer OPVs and for comparison utilize the well‐studied ITIC small molecule NFA.^[15]^ Owing to better energy level alignment of donor and acceptor polymers and excellent spectral response to the indoor lighting sources, CD1:PBN‐10 BHJ demonstrated PCEs of 21.7 and 26.2 % under LED and FL light sources at 1000 lux with high *V*_OC_ of 1.14 V. Till dated, this is the highest reported *V*_OC_ under low light illuminations. On the other hand, the large LUMO offset between CD1 donor polymer and ITIC small molecule acceptor resulted in relatively lower *V*_OC_. CD1: ITIC based IOPVs only exhibit PCEs of 15.4 and 17.9 % under LED and FL light sources, respectively. Furthermore, Yan et al. reported highly efficient IOPVs through interlayer band alignment.^[46]^ As the electron transporting layer (ETL) is critical for obtaining higher device performance, they utilized PDI‐NO and commonly used PFN interlayers and compared the IOPV devices’ performances. The deep HOMO energy level of PDI‐NO can effectively reduce the leakage current and trap‐assisted recombination. They demonstrated IOPVs by utilizing a low bandgap Y6‐O and medium bandgap FTTB‐PDI4 acceptor with PM6 and P3TEA medium bandgap donor materials. Interestingly, with PDI‐NO interlayer, low bandgap acceptor Y6‐O in PM6:Y6‐O BHJ blend demonstrated a PCE of 30.89 %, whereas medium bandgap P3TEA:FTT‐PDI4 BHJ system gave a PCE of 26.67 % under 3000 K LED illumination (1650 lux). Under low light illumination, the IOPVs utilizing PDI‐NO interlayer revealed higher *V*_OC_ compared to PFN that demonstrates the critical role of well band‐aligned interlayer to attain high‐performance IOPVs.

In order to improve the spectral coverage of OPV BHJ, an efficacious strategy is the use of ternary blend system. Through ternary blend system, we can improve the active layer absorbance, morphology and charge transporting properties in OPV devices.[^66^, ^67^, ^68^] So et al. investigated several donor polymers as the ternary components to establish a ternary strategy to enhance the device performance of binary PCDTBT:PC_71_BM BHJ system under 1 sun as well as low light indoor illumination.^[69]^ With a binary BHJ of PCDTBT:PC_71_BM OPV, a PCE of 16.5 % under FL light source at 300 lux was attained. However, with a ternary BHJ of PCDTBT:PDTSTPD:PC_71_BM, the PCE was improved to 20.8 % under FL light source at 300 lux (PCE: 18.9 % under LED light source at 300 lux). The major improved in these devices was the fill factor and current density. The blending of PDTSTPD component passivates shallow traps near the band edges of the BHJ and improve the charge transport properties resulted in enhanced IOPVs device performance. Moreover, Ranbir et al. studied the effect of employing different NFAs as a crystalline modulator in ternary blend system to fabricate efficient IOPVs.^[70]^ They introduced amorphous (PDI2 and PDI4) and semicrystalline (IDT and IDDT) type of NFAs as the third component into OD:PC_71_BM BHJ absorber layer. The optimized ternary blends under indoor light source (1000 lux, LED lamp) demonstrated the PCEs of 21.1, 19.0, 20.5, and 18.2 % for IDT, IDDT, PDI2, and PDI4 respectively, on incorporation of third component in OD:PC_71_BM, whereas the binary host blend only demonstrated a PCE of 14.15 % under indoor light illumination. The addition of semicrystalline IDT among other NFAs enabled the formation of compact nanoscale morphology with well‐dispersion and smaller π‐π stacking distance, thereby resulting in the efficient charge separation, the faster charge carrier transport, the reduced energetic disorder, and the suppression of non‐geminate recombination. Yang et al. adopted the ring‐fusion strategy to design and synthesis the new acceptor materials which can effectively tuned the optoelectronic and structural properties of conjugated materials.^[71]^ The optimal amount of newly synthesized Y−Th2 NFA was introduced as third component into PBDB‐TF(PM6):Y6 to demonstrate a ternary blend system. The ternary blend PM6:Y−Th2:Y6 exhibited a PCE of 16.1 % under standard illumination condition and 22.7 % under indoor illumination at 1000 lux. The cascaded energy level alignment and complementary absorption improve the photovoltaic properties of ternary OPVs.

### 3.2 Interfacial engineering and energy loss of indoor organic photovoltaics

**Figure 5:**
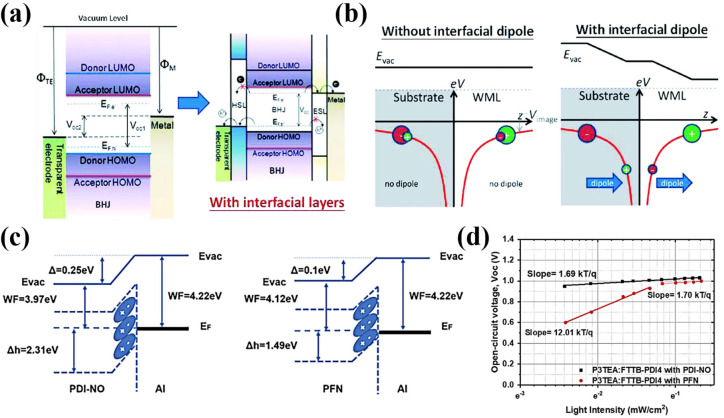
(a) Energy band diagram of typical organic photovoltaics without and with interfacial layers. Reproduced with permission from ref. [74]; copyright 2012, Royal Society of Chemistry. (b) Illustration of work function modification through formation of an interfacial dipole; shifting of the work function is indicated by E_vac_. Reproduced from ref. [75]; copyright 2014, Wiley‐VCH. (c) Energy band alignments of electron‐transporting layer and Al interface, representing the band bending characteristics. (d) Intensity‐dependent V_OC_ of P3TEA:FTTB‐PDI4 with PDI‐NO and PFN interlayers. Reproduced with permission from ref. [46]; copyright 2019, Cell Press.

Organic photovoltaics are assembled from a complex cooperative of layers of different materials, where each layer plays a distinct role within the device. In this section, we will discuss the role of the interfacial layers that bridges the transparent electrodes and the BHJ photoactive layer, and energy loss mechanism in OPV devices under low light illuminations. The energy difference between *E*_F,h_, and *E*_F,e_ under light illumination determines the *V*_OC_ intrinsically (Figure 5a). For realizing enhanced carrier collection and optimum *V*_OC_ of OPV devices, the proper energy level alignment at the electrode/photoactive interface is required. HOMO level of donor and LUMO level of acceptor should be well aligned with the work function (WF) of anode and cathode, respectively.[^72^, ^73^, ^74^] Therefore, the introduction of interfacial layers can be used to modify the WF of the electrode to facilitate barrier‐less Ohmic contact. Besides, the suitable interfacial layer is also sought to improve carrier extraction selectivity by controlling the Fermi level to either *E*_F,h_ of the donor for the hole and the *E*_F,e_ of the acceptor for electron collection (Figure 5a).^[75]^ They can be employed to enhance carrier selectivity at the electrode/photoactive interface. Unwanted carrier recombination can happen at the electrode lowering the device performance as BHJ layer mixed from both donor and acceptor are in direct interaction with the electrodes. However, this unwanted interfacial carrier recombination can be minimalized by employing the interfacial layers with appropriate carrier selectivity, as a result, the device performance can be improved.

Nonetheless, the explanation of energy alignment at the electrode/photoactive layer interface is complicated, as there are numerous reported mechanisms. For instance, the interfacial modification can be explained by electric double layer formation, charge transfer, spontaneous dipole orientation in the bulk, and the spontaneous dipole orientation at the interface.[^46^, ^73^, ^75^, ^76^, ^77^, ^78^] The interfacial layers can alter the interfacial dipole to initiate the vacuum‐level shifting when they are physisorbed on a conducting electrode. The dipole is created at the interface due to the unequal moving ability of positively and negatively charged constituents (Figure 5b). An example of this proposed mechanism is the demonstration of PDI‐NO and PFN as WF modifiers of the Al electrode. Upon Al modification with PDI‐NO, the WF shifts from 4.22 to 3.97 eV, while by employing PFN, the WF shifts from 4.22 to 4.12 eV (Figure 5c).^[46]^ These interfacial layers reduced the WF of Al, thus the Ohmic contact with the LUMO acceptors can be achieved by increasing built‐in‐field in the device. Moreover, the difference between HOMO and Fermi level (Δ*h*) represents the hole extraction barrier of these interfacial layers showing that the PDI‐NO has superior hole‐blocking capability compared to PFN. Also, the effect of the HOMO level of both PDI‐NO and PFN is investigated under indoor lighting illumination. The ideality factor of PFN significantly increases upon decreasing the light intensity from around 1000 to around 200 lux, indicating severe trap‐assisted recombination (Figure 5d). However, the PDI‐NO‐based device has a *kT*/*q* slope closer to unity in the *V*_OC_ as a function of the light intensity plot, indicating lower trap‐assisted recombination and shunt resistance at low light intensity.[^46^, ^79^] At this state, the device with PDI‐NO interfacial layer tends to have low energy (*V*_OC_) loss compared to the PFN‐based interlayer device. These results imply that the PDI‐NO is more suitable to be used as an interfacial layer in the OPV device for low‐light indoor application.

**Figure 6:**
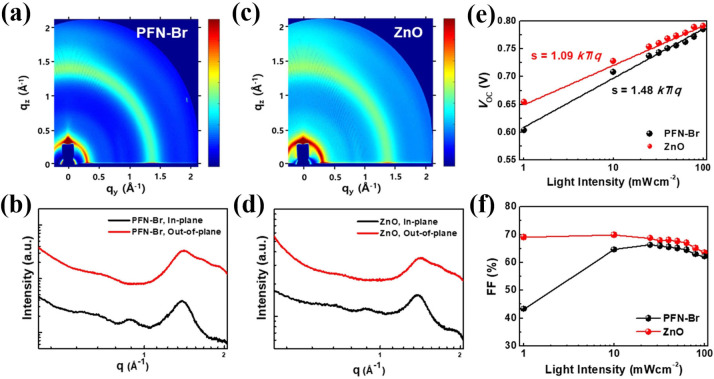
(a–d) 2D GIWAXS patterns and line‐cut profiles of PTB7‐Th:PC_71_BM blend films coated on PFN−Br electron transporting layer (a,b) and ZnO electron transporting layer (c,d). (e,f) Light intensity‐dependent photovoltaic parameters of both devices: (e) V_OC_; (f) FF. Reproduced with permission from ref. [80]; copyright 2020, Elsevier.

Another example is the comparison between PFN and ZnO interfacial layers.^[80]^ The GIWAXS two dimensional (2D) patterns and line‐cut profiles of PTB7‐the:PC_71_BM active film prepared on PFN−Br (Figure 6a,b) and ZnO (Figure 6c,d) showed similar morphology, molecular orientation, and π‐π stacking coherence length of the active layer. However, the different photovoltaic performance is due to the better interfacial contacts between the photoactive layer and ITO in the ZnO OPV. Nam, et.al. measured the photovoltaic properties of PFN−Br and ZnO‐based OPVs under different light intensities from 1 sun to 0.01 sun to elucidate the low light operation of these OPVs. As shown in Figure 6e, the *V*_OC_ values of ZnO and PFN−Br are similar under 1 sun conditions. However, the *V*_OC_ of the PFN‐based OPV drastically declines at low light intensities. It is also found that the FF of the PFN−Br drastically decreases under low light illumination (Figure 6f). These declines imply the high leakage current on the PFN−Br‐based devices.^[80]^ From these results, we can draw conclusions that the interfacial layers for low‐light indoor application should possess high shunt resistance and a low ideality factor to circumvent the *V*_OC_ loss and to sustain high FF at a low light illumination.

Furthermore, to understand the *V*_OC_ loss mechanism under low light illumination (200–1000 lux), we summarized the *V*_OC_ of various IOPVs under low light and compared with *V*_OC_ under 1 sun condition. When the illumination conditions change from 1 sun to indoor low intensity lighting conditions, the *V*_OC_ of OPV devices decreases unavoidably. The *V*_OC_ loss under indoor light illuminations can be derived by the dependence of *V*_OC_ on the light‐dependent photocurrent *I*_ph_ [Eq. 1]:

(1) $$V_{OC}=\frac{nkT}{q}ln\frac{I_{ph}}{I_{o}}$$

where *n* is the ideality factor of the diode, *k* is the Boltzmann constant, *T* is the temperature, *q* is the elementary charge, *I*_ph_ is the photocurrent, and *I*_o_ is the dark current. From Equation (1), we can infer that, under a fixed illumination, the materials with small dark current should give rise to a large *V*_OC_. The change in *V*_OC_ under room lights can be expressed by Equation 2:^[69]^

(2) $$\Delta V=\frac{nkT}{q}ln\frac{I_{ph,\;sun}}{I_{ph,\;room}}$$

where *I*_ph,sun_ and *I*_ph,room_ are the photocurrents under 1 sun and room light illuminations, respectively. Equation (2) demonstrates that Δ*V* is independent of *I*_o_. In general, *I*_ph_ follows power law dependence. Specifically, *I*_ph_ ∝ *P*_light_^*α*^, where *P*_light_ is the incident light power and the exponent *α* is a constant ranging from 0.75 in the case of space‐charge limited (SCL) photocurrent to 1.0 for the space charge free limit.^[81]^ The solar spectrum consists of a wide range of infrared light, which contributes slightly to the PV performance, while LED and FL spectra mostly cover only the visible region, which corresponds to the PV performance. We therefore write *P*_light, Sun_=*P*_Sun_ *θ*, where *P*_Sun_≈100 mW cm^−2^ is the nominal incident power intensity of 1 sun illumination and *θ* is its fraction of light without infrared radiation (<780 nm, defined by the range of *V*(*λ*) corresponding to photopic vision). Then, Equation (2) can be re written as Equation (3):

(3) $$\Delta V\approx \frac{nkT}{q}ln\frac{P_{\;{sun}^{\theta }}}{P_{\;room}}$$

and the *V*_OC_ of room light illumination can be expressed by Equation 4:

(4) $$V_{OC,room}\approx V_{OC,sun}-\frac{nkT}{q}ln\frac{P_{\;{sun}^{\theta }}}{P_{\;room}}$$

**Figure 7:**
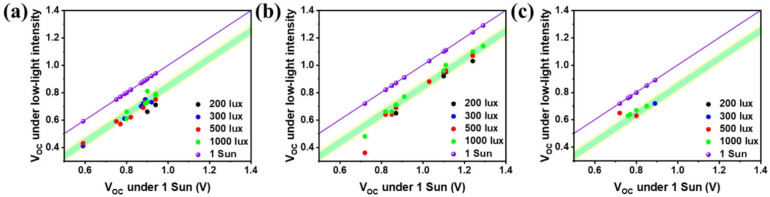
Comparison of V_OC_ under 1 sun illumination and under low light illumination of various BHJ absorber layers: (a) fullerene‐based acceptor materials; (b) nonfullerene‐based acceptor materials; (c) ternary IOPV materials.[^2^, ^8^, ^14^, ^15^, ^17^, ^19^, ^60^, ^61^, ^62^, ^63^, ^64^, ^69^, ^70^, ^71^, ^82^, ^83^, ^84^]

By considering the area under the solar spectrum, 56.65 % of the solar power comes from wavelength less than 780 nm. Using *P*_room_≈56–280 μW cm^−2^ (200–1000 lux) and assuming *n*≈1, a computed energy loss Δ*V*≈0.17–0.14 V, which is almost the same energy loss of experimental reported values shown in Figure 7 for fullerene, nonfullerene and ternary IOPVs. The majority of the reported results satisfy the Equation (4) and illustrates that Δ*V* should be independent of the material choice. Nevertheless, for obtaining higher *V*_OC_ under low light illuminations, it is important to choose BHJ OPV materials that have a large *V*_OC_ under 1 sun illumination.

### 3.3 Large‐area indoor organic photovoltaic modules

**Figure 8:**
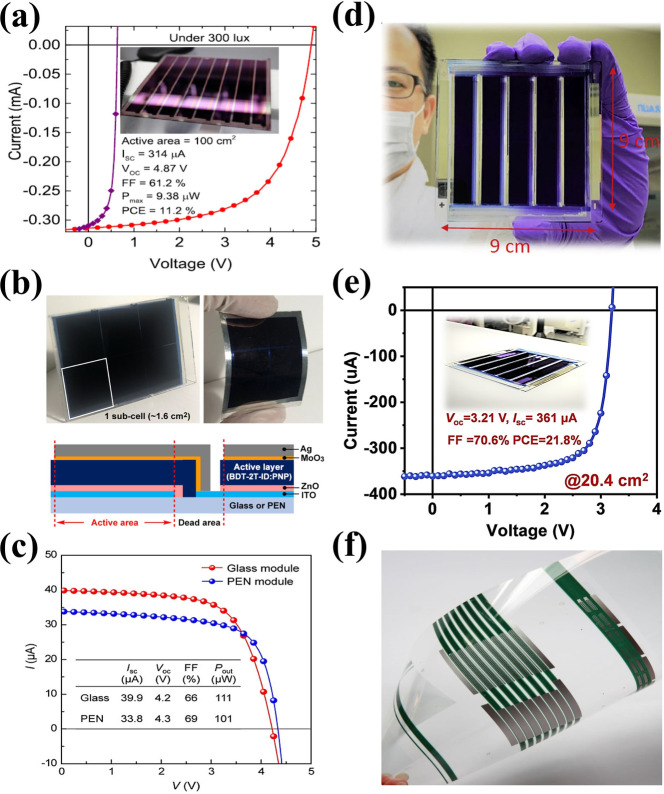
Large‐area organic photovoltaic modules for indoor applications: (a) Current‐voltage characteristics of 100 cm^2^ PCDTBT:PC_71_BM module. The photo of the module is shown in the inset. Reproduced with permission from ref. [14]; copyright 2016, AIP Publishing. (b) Photographs and device architecture of IOPV modules with a BDT‐2T‐ID‐based rigid glass substrate (left) and a flexible PEN substrate (right). (c) Current–voltage characteristics under white LED illumination at 200 lux. Reproduced with permission from ref. [97]; copyright 2019, American Chemical Society. (d) Photograph of TPD‐3F‐based IOPV module and (e) representative current‐voltage characteristics of TPD‐3F‐51 K:IT‐4F module under a fluorescent lamp with an illumination of 1000 lux. Reproduced with permission from ref. [99]; copyright 2020, Cell Press. (f) Fully solution‐processed PV2001:PCBM‐based flexible OPV module and cells by VTT Finland. Reproduced with permission from ref. [100]; copyright 2020, IOP Publishing.

There is no doubt that IOPVs have shown great potential for indoor energy harvesting due to easy bandgap tunability, higher absorbance coefficient and excellent spectral response with emission spectra of indoor lighting systems. Specially, the development of efficient NFAs made possible to obtain higher *V*_OC_ and lower the trap‐assisted recombination for IOPVs. Additionally, solution processability, light weight, flexibility and potentially low cost roll‐to‐roll (R2R) manufacturing make them excellent choice for indoor application.[^24^, ^26^, ^85^, ^86^, ^87^, ^88^] Although, large‐area OPVs are exclusively studied to optimize the large scale manufacturing process through different solvent systems (halogenated, non‐halogenated) and manufacturing techniques (spin‐coating, slot‐die coating, spray coating, 3D printing, ink‐jet printing, R2R, etc.) but there are only few reported about upscaling of OPVs for low‐light or indoor applications.[^89^, ^90^, ^91^, ^92^, ^93^, ^94^, ^95^, ^96^, ^97^, ^98^, ^99^] Harrison et al. fabricated a PCDTBT:PC_71_BM conventional 14×14 cm^2^ OPV module with an active area of 100 cm^2^ for indoor applications and achieved a PCE of 11.2 % generating *P*_max_ of 938 μW under 300 lux FL light source (Figure 8a).^[14]^ Ryota et al. reported a solution processed small‐molecule OPV modules fabricated using a rigid glass substrate and a flexible polyethylene naphthalate (PEN) substrate as shown in Figure 8b.^[100]^ They demonstrated BDT‐2T‐ID:PNP blend based six cells series connected modules with active area of 9.5 cm^2^ which exhibited PCE of approximately 15 % under white LED illumination at 200 lux, delivering an *P*_max_ of 111 μW (Figure 8c). Interestingly, OPV module fabricated on PEN flexible substrate also demonstrated comparable PCE with P_out_ of 101 μW. Similarly, they also reported an outstanding flexible mini‐module (active area: 9.6 cm^2^) with 1DTP‐ID small molecule donor and PNP acceptor materials and obtained a PCE of approximately 17 % generating *P*_max_ of 95.4 μW under 2900 K LED illumination at 200 lux.^[101]^ Liao et al. demonstrated the NFAs based OPV module with absorber layer containing TPD‐3F donor and IT‐4F acceptor materials.^[102]^ OPV modules active layer materials were dissolved in chlorine‐free solvents such as xylene and fabrication process was carried out in ambient environment. TPD‐3F:IT‐4F based IOPV module exhibited an excellent PCE of 21.8 % under FL illumination at 1000 lux (Figure 8d,e).

**Figure 9:**
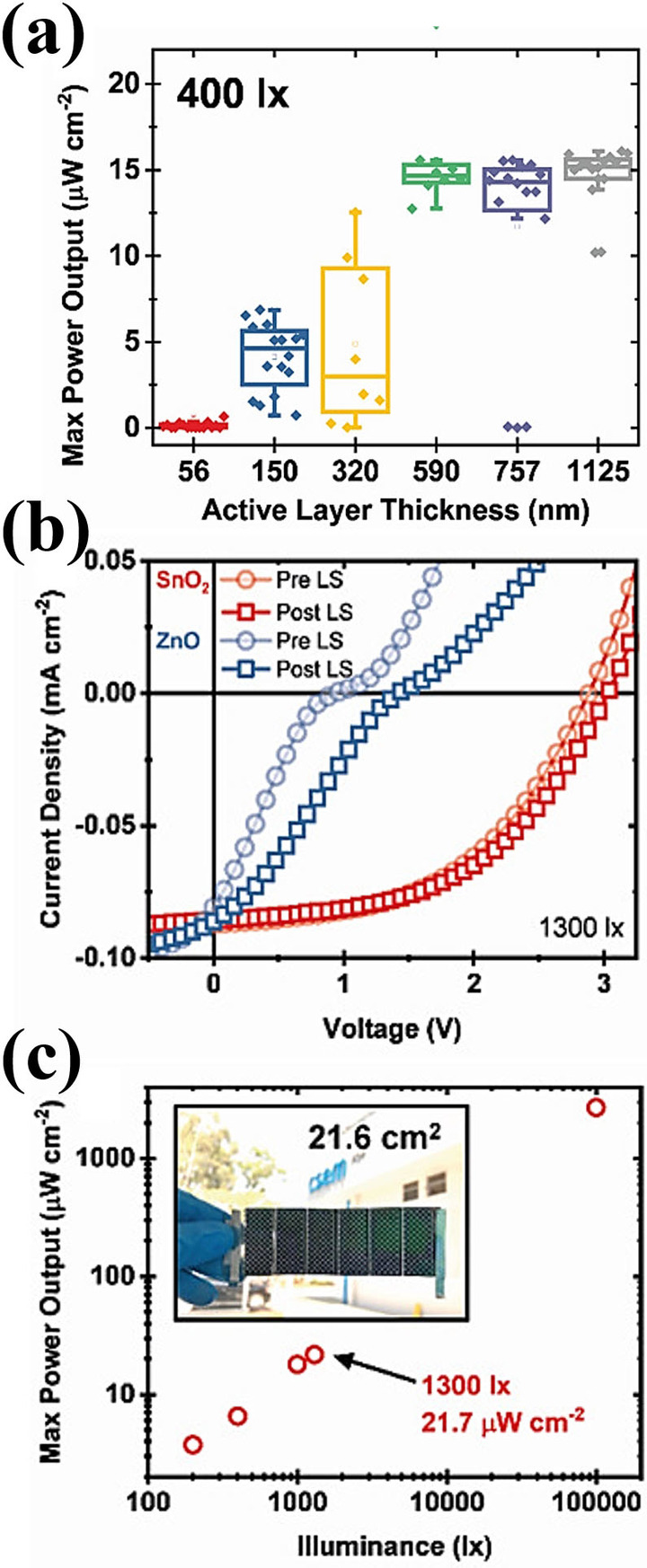
(a) Photoactive layer thickness‐dependent device maximum power output for IMI/ZnO/P3HT:O‐IDTBR/PEDOT:PSS/Ag devices at 400 lux illumination. (b) Current density–voltage characteristics of fully printed 21.6 cm^2^ modules at 1300 lux before and after 1 min of 1 sun light illumination. (c) Light intensity‐dependent performance of the best SnO_2_‐containing module, and photograph of said module (inset). Reproduced from ref. [104]; copyright 2021, Wiley‐VCH.

The OPV module fabrication process adopted in the aforementioned examples was a spin‐coating method, whereas large scale production of OPV modules demands easy printing techniques, such as slot‐die coating or R2R processing. A team of scientists from VTT Technical Research Center in Finland demonstrated the all solution processed OPV modules prepared by R2R slot‐die coating and screen printing on a flexible plastic substrate indium tin oxide (ITO) and compared the device performance of zinc oxide (ZnO) and tin oxide (SnO_2_) electron transporting layer (ETL) under standard illuminations and indoor light conditions.^[103]^ The overlapped spectra of photo‐absorber layer PV2000:PCBM with different indoor light sources was a good match for obtaining relatively higher device performance under FL and LED illumination. At certain lux level, LED has a higher irradiance power than FL source and was observed as an increase in current values. The enhanced current values were also observed in grid structured top electrode cells from fully covered alongside to slightly increased *V*_OC_ and FF. The encapsulated SnO_2_ containing module with grid structured top electrode (Figure 8f) exhibited high indoor light‐harvesting properties with PCE up to 13.4 % in modules. Recently, Luke et al. investigated the low light IOPVs device performance of commercially feasible inverted device architecture, employing all solution processed R2R fabrication technique and relatively low‐cost polymer (P3HT) donor and NFA (O‐IDTBR) acceptor as active layer materials.^[104]^ This is particularly important as thick layers are easier to manufacture by R2R processes (Figure 9a). Their results suggested that screening of possible low light OPV devices using 1 sun illumination is not appropriate, as some OPV devices that perform well at low light intensities may appear to not work well under standard illumination conditions. They also observed and characterized light‐soaking effect with ZnO and SnO_2_ ETLs that can seriously affect the IOPV device performance (Figure 9b). In general, flexible OPV devices with ZnO ETL exhibit s‐shape current density‐voltage (*J‐V*) characteristics due to the absence of UV light region in most of the flexible substrates. The devices are limited by poor charge extraction at the ETL/active layer interface when ZnO ETL is used due to its high density of sub‐gap trap states. Two clear strategies for avoiding light soaking was demonstrated that overcome this poor charge extraction: replacing the ZnO ETL with SnO_2_ nanoparticles or by employing an acceptor with a sufficiently shallow LUMO level to avoid any electron extraction barrier. Under low light illuminations the active layer thickness become insensitive as thick active layer not only reduce the dark current but also easier to fabricate by R2R process. Fully printed light‐soaking free large area module (Figure 9c) achieved a *P*_max_ of 21.7 μW cm^−2^ by utilizing SnO_2_ ETL, under LED light illumination at 1300 lux whereas the OPV modules with ZnO ETL exhibit S‐shaped *J‐V* curves even after light soaking.

### 3.4 Device stability of indoor organic photovoltaics

In recent years, the development of high efficiency photo absorber materials and innovative fabrication techniques that are compatible with large scale, low cost manufacturing process, demands the attentions to be focus on operational stability of OPVs for commercialization. Numerous efforts have been devoted to understand the degradation mechanism of OPVs under standard simulated illumination (AM 1.5G, 100 mW cm^−2^) condition or outdoor in direct sunlight, whereas there are few reported investigations into the degradation behavior of OPVs in indoor low light environments.[^25^, ^105^, ^106^, ^107^, ^108^, ^109^] However, relatively mild indoor conditions, compared to the severe outdoor or intense stability evaluation conditions such as heat, solar irradiation, and fluctuating weather, can provide a pathway for prolonged device operational stability. In this section, we will discuss the recently reported IOPVs stability assessment investigations and prospective on their degradation mechanism under low light illumination conditions.

Most of the donor and acceptor materials are sensitive to light and oxygen, the photo‐oxidation process of the photoactive materials can cause rapid degradation of device performance with the formation of superoxide radical ions, which are found to be the major degradation pathway and strongly influenced by the LUMO energy level of the acceptor materials. This degradation mechanism is both applicable for fullerene and nonfullerene based BHJ and can be avoided to some extent by designing the acceptor materials with sufficient electron affinities.[^71^, ^110^, ^111^, ^112^]

**Figure 10:**
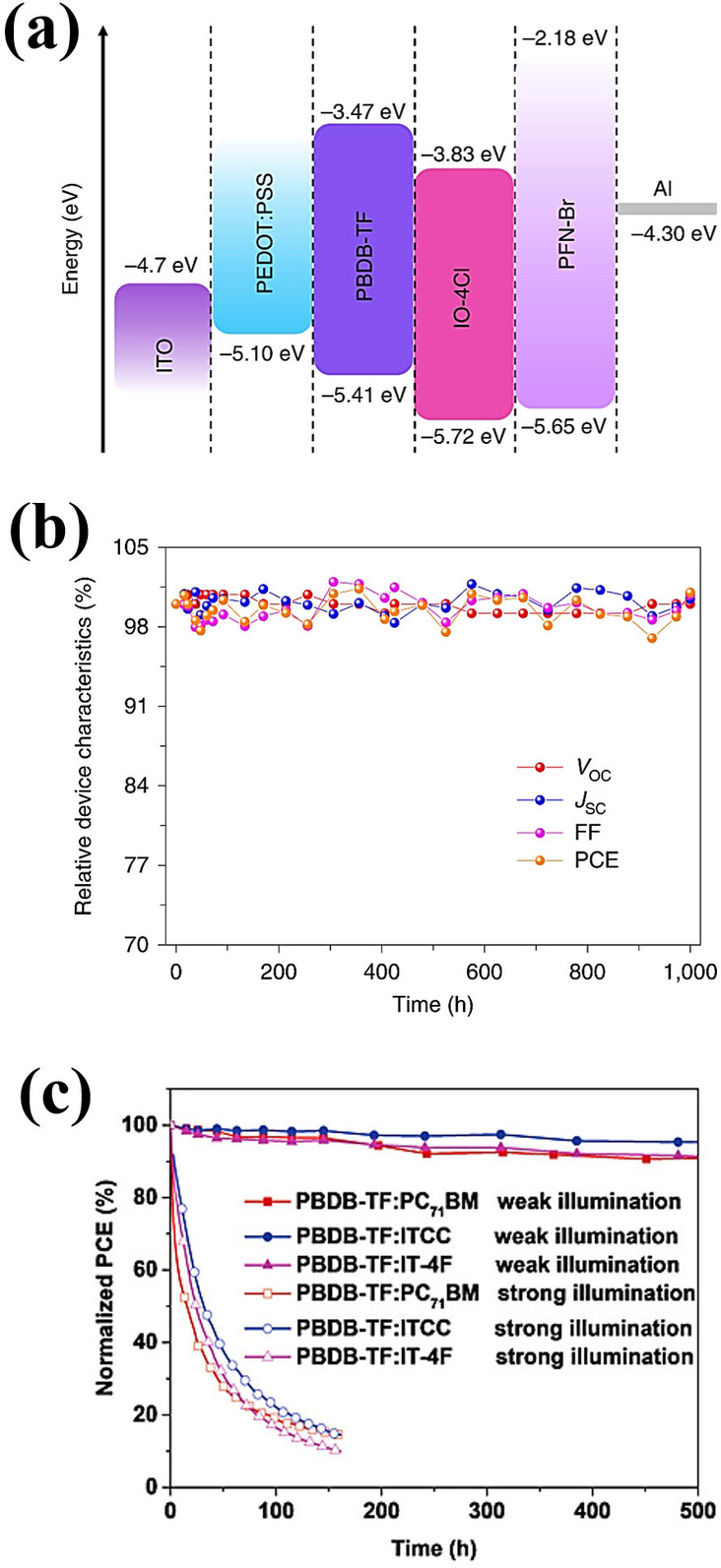
(a) Energy band diagram of conventional indoor organic photovoltaics. (b) Relative photovoltaic characteristics vs. time. Reproduced with permission from ref. [2]; copyright 2019, Nature Publishing Group. (c) Stability trends of the three different organic photovoltaic devices under continuous weak and strong illumination. Reproduced from ref. [19]; copyright 2020, Wiley‐VCH.

**Figure 11:**
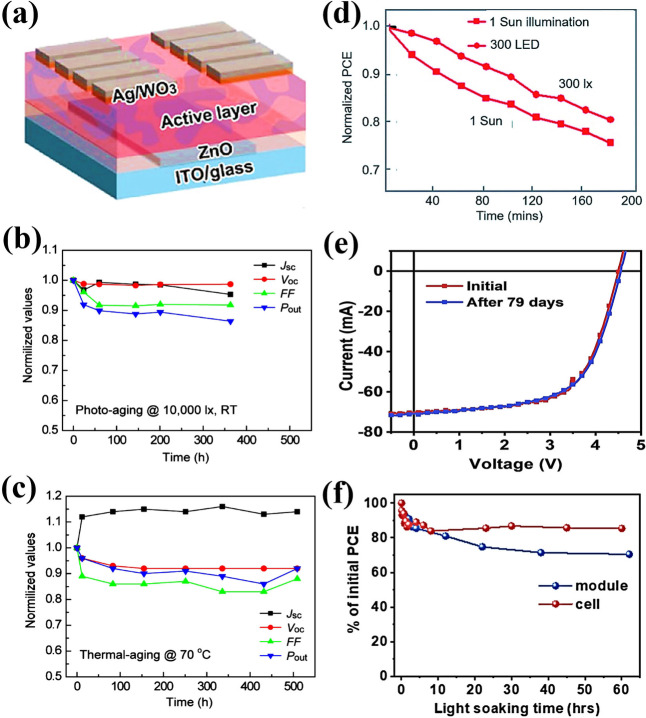
(a) Schematic representation of indoor BHJ OPV devices with an inverted configuration. (b,c) Changes in normalized J_SC_, V_OC_, FF, and P_out_ for the OPVs based on BDT‐2T‐ID:PNP as a function of photo‐aging time under white LED illumination at 10000 lux at room temperature (b) and thermal aging at 70 °C under dark (c). Reproduced with permission from ref. [100]; copyright 2019, American Chemical Society. (d) A comparison of degradation behavior of OPV under 1 sun and LED illumination. Reproduced with permission from ref. [69]; copyright 2018, Royal Society of Chemistry. (e,f) OPV module stability under dark and continuous light illumination. Reproduced with permission from ref. [102]; copyright 2020, Cell Press.

One effective way to avoid the oxygen‐induced photochemical degradation of the photoactive layer is encapsulation of the OPV device within glass or plastic films. However, glass films are not suitable for flexible OPVs and most of the plastic films used for encapsulation cannot fully block oxygen diffusion. Therefore, the development of suitable encapsulation materials for both rigid and flexible OPV devices is still a challenge that need to be address. Recently, Hu et al. reported a chemical reaction between low work function interlayer such as PEI or PEIE with nonfullerene based OPVs.^[113]^ Where, amine interfacial material reacts as a nucleophile with the C=O moiety of the ITIC that damages the original electronic structure and the intramolecular charge transfer of the ITIC molecules. Similarly, the interfacial chemical reaction of hole transporting layer (HTL) with photoactive layer cause rapid drop of device performance of OPVs. Although, such interfacial reaction mechanisms can be stopped through effective passivation of metal ion doping or interfacial modification, the development of suitable interlayers with excellent performance and stability are prerequisite for the progress of high‐performance and stable OPV devices.[^104^, ^114^, ^115^] Cui et al. demonstrated the device stability of PBDB‐TF:IO‐4Cl based conventional OPV cell under continuous indoor light illumination (Figure 10a,b).^[2]^ The OPV cells maintained their initial PCE after 1000 h that exhibits a great potential of OPVs for indoor applications with excellent device performance and operational stability. In another report, they also demonstrated the operational device stability of PBDB‐TF donor with PC_71_BM, ITCC and IT‐4F acceptor materials under continuous strong and weak light illumination with varied temperature conditions (Figure 10c).^[19]^ All devices were encapsulated to block water and oxygen. Under weak illumination conditions with a temperature of 25–30 °C, all three OPV devices maintained 95 % of their initial PCEs. However, all three OPV devices exhibited a fast degradation under strong light illuminations. Similarly, Arai et al. demonstrated the operational stability of BDT‐2T‐ID:PNP based IOPVs under 10000 lux light illumination and at 70 °C under dark conditions as illustrated in Figure 11a–c.^[100]^ The IOPV devices under continuous light illumination sustain about 90 % of their original PCE, whereas the devices with thermal aging exhibited about 95 % of their initial PCE. Yin et al. compared the unencapsulated PCDTBT:PC_71_BM OPV device stability under standard 1 sun (AM 1.5G, 100 mW cm^−2^) and indoor LED lamp (300 lux) in air as shown in Figure 11d and observed that the OPV devices under low‐light illuminations exhibited similar degradation kinetics to those under standard 1 sun illumination, with slightly lower degradation rate.^[69]^ These results suggested the relatively weak dependence of photochemical degradation on the light intensities. To date, most of the stability studies under low light illumination conditions are based on small‐area single‐cell devices, whereas, for real application, OPV modules with reasonable device areas that provide sufficient power for the working of IoT devices are required. Therefore, the evaluation of OPV module stability under indoor environment is preferable. Liao et al. studied the TPD‐3F‐51 K:IT‐4F OPV modules stability under dark conditions and compared the device stability of single cell and module under standard illumination conditions (Figure 11e,f).^[102]^ The results indicated that the module under dark conditions retained >98 % of the initial PCE, whereas single cells and modules under standard illumination conditions exhibited typical burn‐in behaviors with decreases in both *J*_sc_ and *V*_OC_ and a larger drop in FF after 20 h of light soaking. Thereafter, the PCE stabilized to around 70 % for the module and around 85 % for the cell. The stability data of single cell and module indicates that the losses in the large area module could be higher than those in the small cell size, owing to a higher probability of defects. Therefore, it is essential to evaluate the stability of large‐area modules under illumination conditions to realize the true potential of OPVs for indoor application.

## 4 Prospects to Improve Indoor Organic Photovoltaic Performance

IOPV device design is undoubtedly a challenging task, as the device optimizations carried out under standard illumination conditions could alter under low light illumination, leading to the poor device performance. Also, the different light illumination sources with nonuniform light intensities and spectral response could affect the device performance significantly. In this section, we will discuss various approaches to improve the device performance of IOPVs through light management that can reduce the effect of nonuniform and directional dependency of IOPVs under low light illumination conditions.

### 4.1 Semitransparent indoor organic photovoltaics

**Figure 12:**
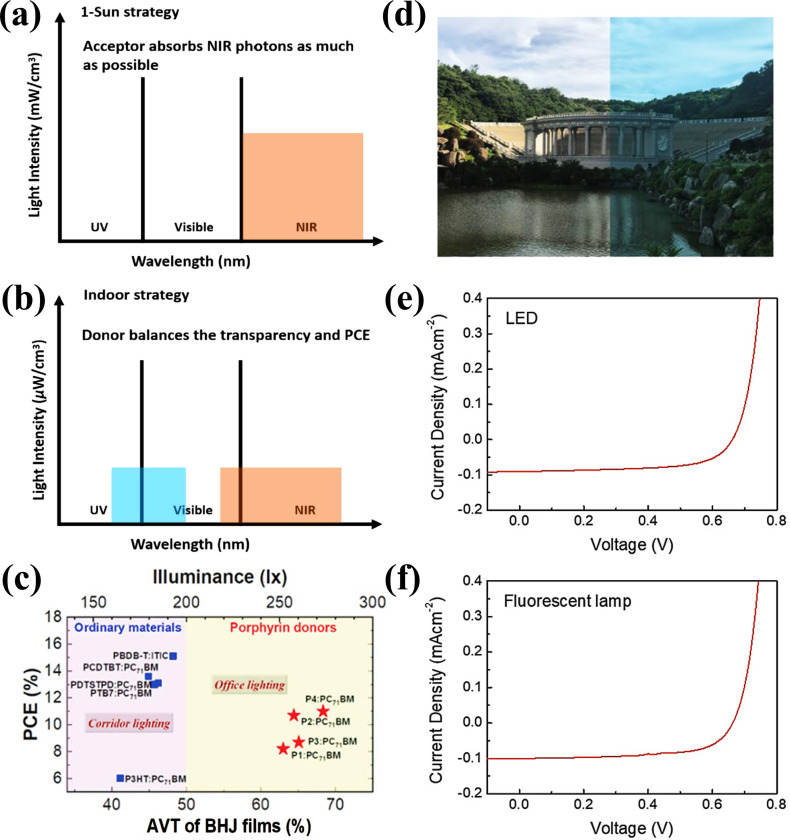
(a,b) Illustration of the strategies for semitransparent organic photovoltaics under 1 sun (a) and indoor (b) conditions. (c) Device performance vs. average transmittance of selected typical polymer:acceptor‐ (blue) and porphyrin:fullerene (red)‐based photovoltaics. Reproduced from ref. [116]; copyright 2020, Wiley‐VCH. (d) A photograph taken without (left) and with (right) filtering by a quaternary blend film. (e,f) Current density‐voltage characteristics of semitransparent quaternary photovoltaics with Ag electrode of 15 nm under 1000 lux LED (e) and 1000 lux fluorescent lamp (f). Reproduced from ref. [117]; copyright 2019, Wiley‐VCH.

The donor and acceptor materials utilized for IOPVs are good absorber in the visible region which appears to be dark in color and the presence of such dark objects for indoor application could affect the visual senses of human beings. In particular, public places such as classrooms, hospitals, supermarkets, or homes, demand not only high performance but also pleasing appearance through colorful designs and patterns for adorning the indoor environment. Although, there are only few examples of semitransparent IOPVs but the semitransparent OPVs have the potential to fulfil such requirements along with high performance under low light environments. Yin et al. reported a comprehensive study about highly transparent and true colored semitransparent IOPVs.^[116]^ A common strategy for high‐performance semitransparent OPVs under 1 sun condition is that the OPV device with thin photo‐absorber layer is fabricated for reasonable transparency in the visible region and for the compensation of energy loss due to the transmission of visible photons, a NIR absorbing acceptor material is utilized in BHJ as shown in Figure 12a. Through this strategy, a semitransparent OPV with sufficiently high PCE can be obtained. However, this strategy cannot be adopted for IOPVs, as most indoor light sources have emission in the visible region and BHJ with NIR absorbance can have serious spectral mismatch, resulting in significant energy losses under indoor environment. The majority of efficient IOPV donor/acceptor materials will appears dark, even with transparent electrodes, owing to their strong absorbance in the visible region. Yin and co‐workers proposed that porphyrin‐based donor materials can be promising candidate for efficient semitransparent IOPVs. Porphyrin‐ based donors materials have unique absorption profile that have high transmission in the visible region of the most human visual sensitivity and stronger absorption at the blue‐/red‐ends for photovoltaic effect (Figure 12b). Such design allows for photovoltaic applications of high visible transmittance and good color rendering under indoor cold light sources. Through this strategy, they obtained PCE of 10.7 % under 300 lux LED light illumination with average transmittance of 65 %. The PCEs vs AVT of BHJ film comparison of commonly used donor materials for IOPVs and a special class of porphyrin‐based donor materials for semitransparent IOPVs is shown in Figure 8c. Nam et al. demonstrated semitransparent quaternary IOPVs for indoor applications.^[117]^ The average visible transmittance was 48.6 % for the quaternary photoactive blend film and 13.7 % for the semitransparent quaternary OPV device with Ag of 15 nm. The semitransparent quaternary OPV device exhibited a PCE of 13.36 % and 12.94 % under LED and FL light illuminations respectively, at 1000 lux (Figure 12d–f). The PCEs of semitransparent IOPVs can be further improved through optimizing transparent electrode materials and active layer components.^[118]^

**Figure 13:**
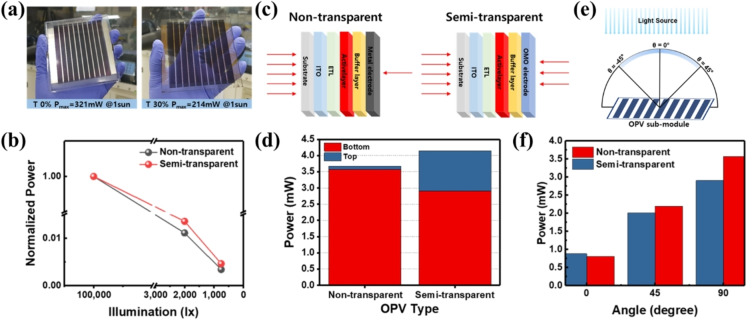
(a) Photographs of nontransparent and semitransparent organic photovoltaic modules (10×10 cm^2^). (b) Normalized power change of nontransparent and semitransparent organic photovoltaic modules at various light intensities (1 sun to 750 lux). (c) Schematic representation of the nontransparent and semitransparent organic photovoltaic device structures and bifacial absorption of light. (d) Bifacial power of nontransparent and semitransparent organic photovoltaic modules under low‐light illumination (2000 lux). (e) Schematic representation of the angle‐dependence measurements. (f) Power change of nontransparent and semitransparent OPV modules with various incident light angles (angle dependence) under low‐light illumination (2000 lux). Reproduced with permission from ref. [5]; copyright 2019, Bentham Science Publishers.

In our previous report, we demonstrated the semitransparent OPV modules having MoO_3_/Ag/MoO_3_ (OMO) electrode and compared the device performance with nontransparent OPV modules under low light illumination conditions.^[5]^ The purpose of this study was to elaborate the potential of semitransparent OPVs for indoor application under non‐uniform and angle dependent illuminations. Figure 13a exhibits the nontransparent and semitransparent (10×10 cm^2^) OPV modules with an active area of 40 cm^2^. Under standard illumination conditions, the nontransparent and semitransparent OPV modules demonstrated *P*_max_ of 321 mW and 214 mW, respectively. However, under low light intensities, 2000 lux and 750 lux, the semitransparent OPV module exhibited relatively higher output power (Figure 13b). The difference is clear, as under low light intensity, all the light is absorbed by the active layer and there is a negligible light reflection from the nontransparent silver electrode and re‐absorption in the active layer. In the case of semitransparent OPV modules, there is a direct light absorbance from the ITO/glass substrate side as well as indirect/reflected light absorbance from semitransparent OMO electrode side that improves the overall output power of semitransparent OPV module under low‐light illumination as compared to nontransparent OPV module. The phenomena of light absorbance in nontransparent and semitransparent OPV modules is illustrated schematically in Figure 13c and the contribution of the output power under 2000 lux light intensity with respect to the OPV module illumination side are illustrated in Figure 13d. In the case of light illumination from substrate side (bottom), the output power of nontransparent OPV module is higher than semitransparent OPV module. However, in the case of light illumination from the electrode side (top), the output power of semitransparent OPV module is higher. We compared output power of both type of OPV modules after the sum of top and bottom sides and found that the power of semitransparent OPV module is higher than nontransparent OPV module under low illumination (Figure 13d). To further investigate the potential of nontransparent and semitransparent OPV modules for indoor light applications, we measured the output power of both types of modules with respect to the incident light angle under a light intensity of 2000 lux (Figure 13e,f). On direct light illumination (*θ*=90°), the nontransparent OPV module shows higher output power than semitransparent OPV module, whereas this difference reduces significantly at an angle of 45° and semitransparent OPV module outperforms at an angle of 0° where no direct light is illuminated to the OPV module. These experimental results suggest that the semitransparent photovoltaics could perform better compared to nontransparent photovoltaics under indoor environment, especially when the photovoltaic devices are not directly illuminated by the indoor lightening system.

### 4.2 Light‐trapping management approaches for indoor organic photovoltaics

Indoor lighting environment is a non‐uniform system where the intensities of light are different at each measurement point with respect to distance and incident angle from light source. Such a non‐uniform environment can limit the device performance of IPVs. To overcome this challenge, the researchers need to focus on the adoption of light‐trapping management techniques such as anti‐reflection coatings, surface texturing, surface plasmonic effect etc., for IPVs. In recent years, many light management concepts which are easily applicable for OPVs has been proposed, most of which are originated from inorganic photovoltaics. In this section, we will discuss various light management approaches which can be utilized to enhance the device performance of IOPVs.

**Figure 14:**
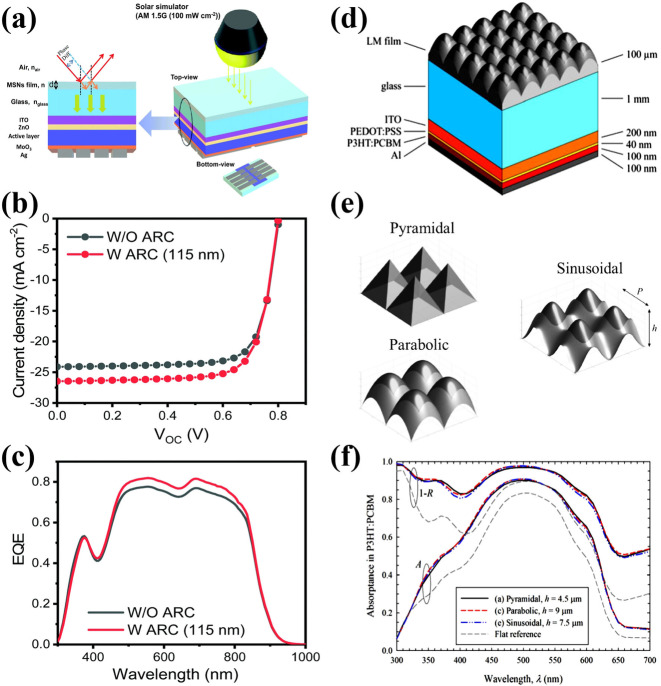
(a) Schematic representation of the antireflection coating on the glass substrate of organic photovoltaics to improve transmittance and device performance. (b) Current density–voltage characteristics and (c) external quantum efficiency spectra of PBDB−T‐2F:BTP‐4F devices with and without the antireflection coating. Reproduced with permission from ref. [119]; copyright 2019, Royal Society of Chemistry. (d,e) Structure of an organic photovoltaic deposited on glass. The microtextured light management film is applied on top of the glass substrate (here shown for the case of parabolic texture) and three types of microscale surface textures: pyramidal, parabolic, and sinusoidal. (f) Absorbance and inverted reflectance (1−R) spectra of organic photovoltaics with three optimal surface textures. Reproduced with permission from ref. [120]; copyright 2014, IEEE.

The fabrication of an anti‐reflection coating (ARC) onto the transparent substrate side of the photovoltaic devices can effectively enhance the light absorbance, resulting in improved device performance. Wang et al. synthesized mesoporous structured silica nanoparticles (MSN) grafted with hexamethyldisiloxane (HMDS) to produce HMDS‐MSN mixtures.^[119]^ After mixing with silica oligomer binder, it can be applied on the glass substrate to fabricate ARC film. With ARC, the transmittance of the glass substrate increases from 90 to 95 %, whereas a drop‐in reflectance from 8 % to 4 % was observed. To increase the light absorbance of OPVs photoactive layer, these ARCs were casted on the outside of the glass substrate of OPV devices. With a 115 nm ARC, the *J*_SC_ and PCE of PBDB−T‐ 2F: BTP‐4F based OPVs increased from 25.2 mA cm^−2^ and 15.4 % to 26.7 mA cm^−2^ and 16.2 % (Figure 14a–c). These results suggest that an ARC can play an important role to enhance the device performance of OPVs. This simple approach can be adopted for IOPVs to reduce the incident light reflection and to further enhance the device performance. Moreover, microtextured light‐management films can be easily fabricated and laminated on top of flat OPV devices or modules. This concept presents a cost efficient and promising solution. However, the shape and size of micro‐textures should be considered carefully to obtain maximum device performance. Lipovsek et al. employed optical simulations to investigate light trapping potential of micro textured (pyramidal, parabolic, and sinusoidal) films laminated on top of the front glass substrate in OPVs and to determine their optimal surface morphology.^[120]^ The results revealed that the OPV devices with pyramidal, parabolic and sinusoidal textures exhibited more than 20 % increase of *J*_SC_ as compared to nontextured OPV devices (Figure 14d–f). The pyramidal textures are highly sensitive to the dimension and incident angle of illumination and might not be appropriate for applications under varying angular illumination conditions. However, parabolic and sinusoidal textures can perform well in a broader range of texture constraints and incident illumination angles. Consequently, ARC and surface texturing approaches can be useful to achieve light trapping and reduce light reflectance. Furthermore, internal reflection from the back electrodes of the OPV can increase the length of the absorption path in the OPV devices and the total light absorption. Hence, these techniques can enable higher performance of OPVs with weak and non‐uniform light intensity.

**Figure 15:**
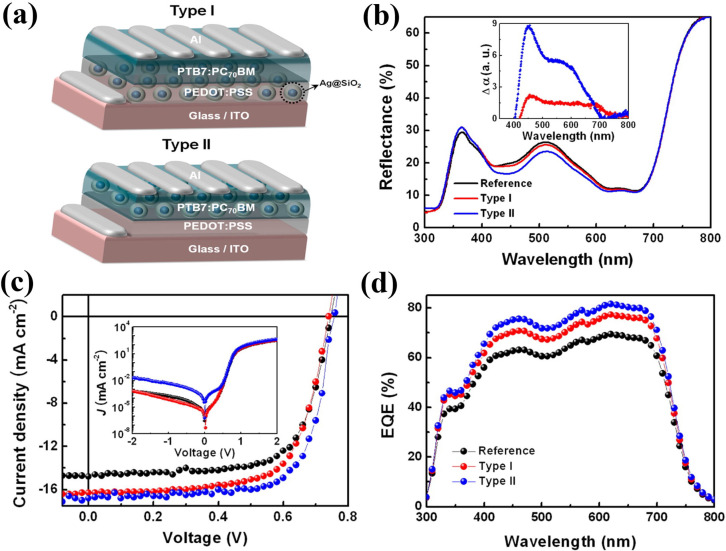
(a) Device structures and (b) reflectance spectra of PTB7:PC_70_BM‐based organic photovoltaics with different spatial locations of Ag@SiO_2_. The inset shows the absorption enhancement (Δα) caused by Ag@SiO_2_. (c) Current density‐voltage characteristics and (d) external quantum efficiency of organic photovoltaics with type I and type II architectures. Reproduced with permission from ref. [121]; copyright 2013, American Chemical Society.

Another exciting method for attaining light trapping in OPVs is the use of metallic nanostructures that support surface plasmons. Through appropriate optimization and engineering of these metallo‐dielectric structures, light trapping can be achieved by scattering at the surface of OPVs, excitation of localized surface plasmon in metal nanoparticles embedded in active layer and excitation of surface plasmon polaritons at the metal semiconductor interface, resulting in an increase of light absorption. Ideally, the light‐absorbing layers of OPVs must be thick enough to absorb incident lights completely. However, most of the OPVs absorber layers have thickness of few hundred nanometers. Therefore, in OPV devices, plasmonic structures can offer great prospects to enhance the light absorbance. Most of the reports incorporate nanoparticles into active layer. However, there have been significant concerns about uniform dispersion of metal nanoparticles into active layer and device performance caused by exciton quenching. For example, Choi et al. incorporated metal nanoparticles at the interface of indium tin oxide (ITO) and PEDOT:PSS or embedded into PEDOT:PSS layer and suggested that to maximize the surface plasmon resonance effect on device performance, it is essential to control the distance between metal nanoparticles and active layer. They demonstrated high‐performance OPVs through incorporation of multi‐positional silica coated silver nanoparticles (Ag@SiO_2_; Figure 15a). The silica shell in Ag@SiO_2_ preserves the surface plasmon resonance effect of the Ag nanoparticles by avoiding oxidation of the Ag core under ambient conditions and also eliminates the concern about exciton quenching by evading direct contact between Ag cores and the active layer. The multi‐positional property refers to the ability of Ag@SiO_2_ nanoparticles to be introduced at both ITO/PEDOT:PSS (type I) and PEDOT:PSS/active layer (type II) interfaces in polymer: fullerene based BHJ OPVs due to the silica shells. The type II structure shows strong light absorption and scattering via enhanced electric field distribution compared to the type I structure, resulting in remarkable enhancement in *J*_SC_ and thus PCE (Figure 15b–d).

**Figure 16:**
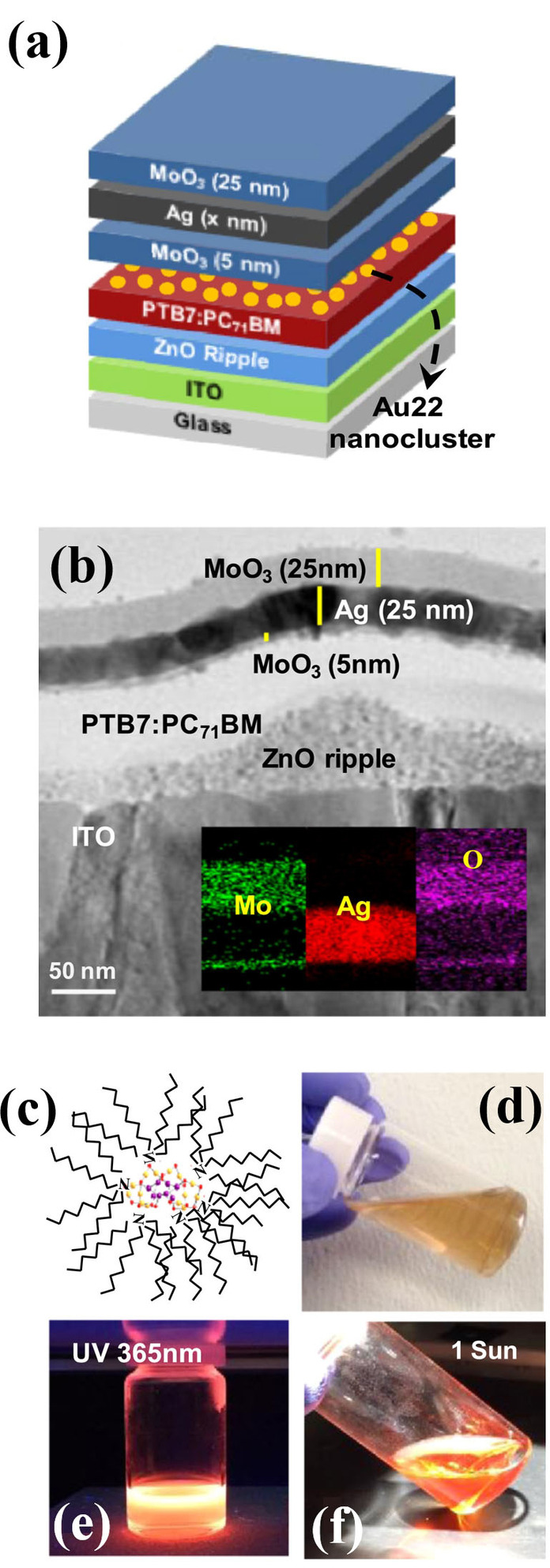
(a) Schematic representation of the device structure. (b) Cross‐sectional TEM images and (c) schematic representation of tetraoctylammonium monolayer‐protected Au22‐QCs. (d) Au22‐QCs solution dispersed in CB. (e,f) PL emission of Au22‐QCs under 365 nm UV irradiation and one sun solar simulator irradiation showing illumination angle dependence of semitransparent solar cells. Reproduced with permission from ref. [122]; copyright 2018, Elsevier.

**Figure 17:**
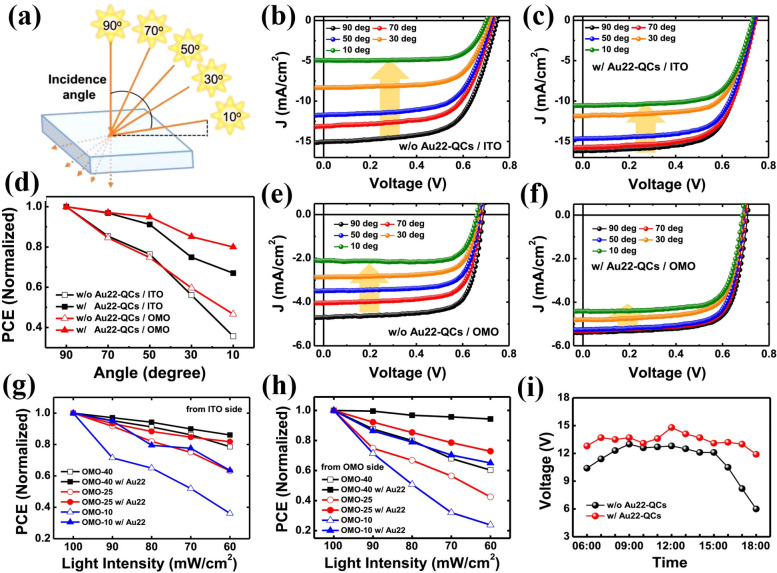
Effect of Au22‐QCs on semitransparent solar cells: (a) Schematic representation of the angle‐dependent PCE measurements. (b,c) J‐V characteristics of the semitransparent solar cell without and with Au22‐QCs with illumination from the ITO side. (d) Normalized PCE change according to the illumination angle of solar simulator light incident from the ITO side or OMO side. (e,f) J‐V characteristics of the semitransparent solar cell without and with Au22‐QCs with illumination from the OMO side. (g,h) PCE change according to the illumination intensity of solar simulator light incident from the ITO side and with different thickness from the OMO side. (i) Out‐lab testing of semitransparent minimodules: Voltage variation of the minimodule measured with one‐hour intervals from 6a.m. to 6p.m. Reproduced with permission from ref. [122]; copyright 2018, Elsevier.

In our previous report, we demonstrated efficient semitransparent OPVs with significantly improved performance at low‐intensity illumination through incorporation of emissive quantum clusters (QCs) consisting of 22 Au atoms.^[122]^ The addition of Au22‐QCs in the semitransparent OPVs resulted a significant reduction of incident light intensity dependence as well as enhanced device performance (Figures 16 and 17). In case of Au22‐QCs incorporated semitransparent OPVs, there was only a 15 % drop in PCE with a reduced light intensity (1.0 sun to 0.6 sun), whereas the PCE of the control device was reduced by around 35 %. Furthermore, outdoor performance using semitransparent mini‐modules confirmed the effect of Au22‐QCs even under real sunlight. The OPV mini‐modules with incorporation of Au22‐QCs exhibited excellent performance with reduced sensitivity to daylight intensities over a day. This reduced sensitivity can be beneficial for building integrated PVs and for indoor low light intensity applications. The angle of incident light can strongly influence the OPV device performance. However, Au22‐QCs incorporated OPVs demonstrated excellent device performance with respect to change in angle of incident light intensity. However, for the OPV devices without Au22‐QCs, *J*_SC_ was reduced by around 70 % on changing angle of incident light from 90° to 10°.

In contrast, the OPVs with Au22‐QCs exhibited only 35 % drop in *J*_SC_ with angle of incident light from 90° to 10°. Moreover, less dependence on the angle of incidence light also offered a great advantage for IOPVs applications. Therefore, it is noticeable that the overall features of Au22‐QCs incorporated OPVs related to the improved performance under low light intensities could be beneficial for practical indoor applications using semitransparent OPVs or general nontransparent OPVs.

## 5 Low‐Power IoT Devices and Potential of Indoor Organic Photovoltaics

**Figure 18:**
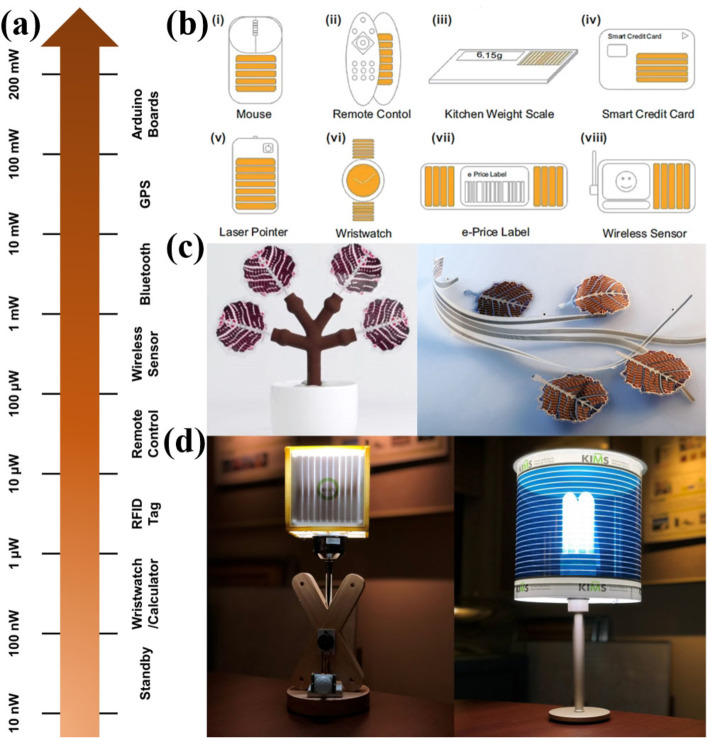
(a) Power requirements of various IoT systems. (b) Photovoltaic device‐integrated low‐power electronic products. Reproduced with permission from ref. [5]; copyright 2019, Bentham Science Publishers. (c) Photographs of fully printed solar trees and leaves by VTT Finland designed for indoor applications (Source: https://blog.drupa.com/de/printed‐trees). (d) Photographs of rigid OPV module and semitransparent flexible module mounted lamps for indoor application to recycle the indoor lights for IoT applications.

Low power IoT devices integrated with photovoltaics have great prospects for health‐care, environmental monitoring, smart homes, smart markets and industry.[^6^, ^123^, ^124^, ^125^] The concept of IoT is beyond the limits where everything is connected for communication purpose with everything. To meet the growing demands of product integrated photovoltaics for indoor applications, the researchers need to focus on the design and development of ultra‐low power IoT products and highly efficient IPVs. To date, most of the IoT products energy requirements are much higher than the energy generation of IPVs. Figure 18a shows the power requirements of different low power IoT devices. The OPV module with active area of 40–50 cm^2^ can only produce *P*_max_ values of 2–3 mW under low light illumination. This power generation might be enough to support or prolong the battery life of indoor IoT systems but for self‐sustaining and battery independent indoor IoT systems, further development for high‐performance IOPVs are much needed. Figure 18b shows the schematics of some basic commercially available PV integrated devices. Such devices (wristwatch, smart cards, e‐price label, remote control, and weighting machine etc.) mostly work independently and are not connected with external device, whereas computer mouse or wireless seniors can be connected to the external systems through Bluetooth or passive Wi−Fi connections. Therefore, the devices integrated with relatively high‐power consumption components such as Bluetooth or Wi−Fi required higher power to work efficiently. Commercially available low power IoT devices with Bluetooth or Wi−Fi connectivity required approximately 2–10 mW input power. It is worth noticing that IOPVs can generate high voltages whereas the major obstacle to operate efficiently such IoT devices are the low currents of IOPVs. One way to overcome this obstacle is voltage and current management through the appropriate design of IOPV modules and electric circuits to fulfil the optimum requirements for IoT systems. Moreover, we can expand the IOPVs active area through different designs to attain higher powers, as OPVs are well known for the flexibility of their designs and patterns, owing to easy solution processability with different fabrication techniques, such as 3D printing and R2R manufacturing. Figure 18c exhibits the beautiful design of solar tree designed by a group of researchers from VTT Finland for indoor applications. Such applications not only can produce higher indoor power but can also give pleasant appearance for indoor ornament. Similarly, the table lamps shown in Figure 18d can recycle the lamp light to produce relatively higher powers for IoT devices. Although, indoor IoT systems have the motivation of ultra‐low power consumptions but these applications are not just limited to few sensors such as motion, temperature, humidity etc. the concept of IoT devices is beyond limits of a closed space to an ecosystem which benefits the human life through the environment wherein smart services are provided to utilize every activity anywhere and anytime. To fulfil the requirements of such systems, the researchers need not only to design ultra‐low power IoT system but also provide better energy management through creating efficient indoor energy harvesting systems by utilizing IOPVs.

**Figure 19:**
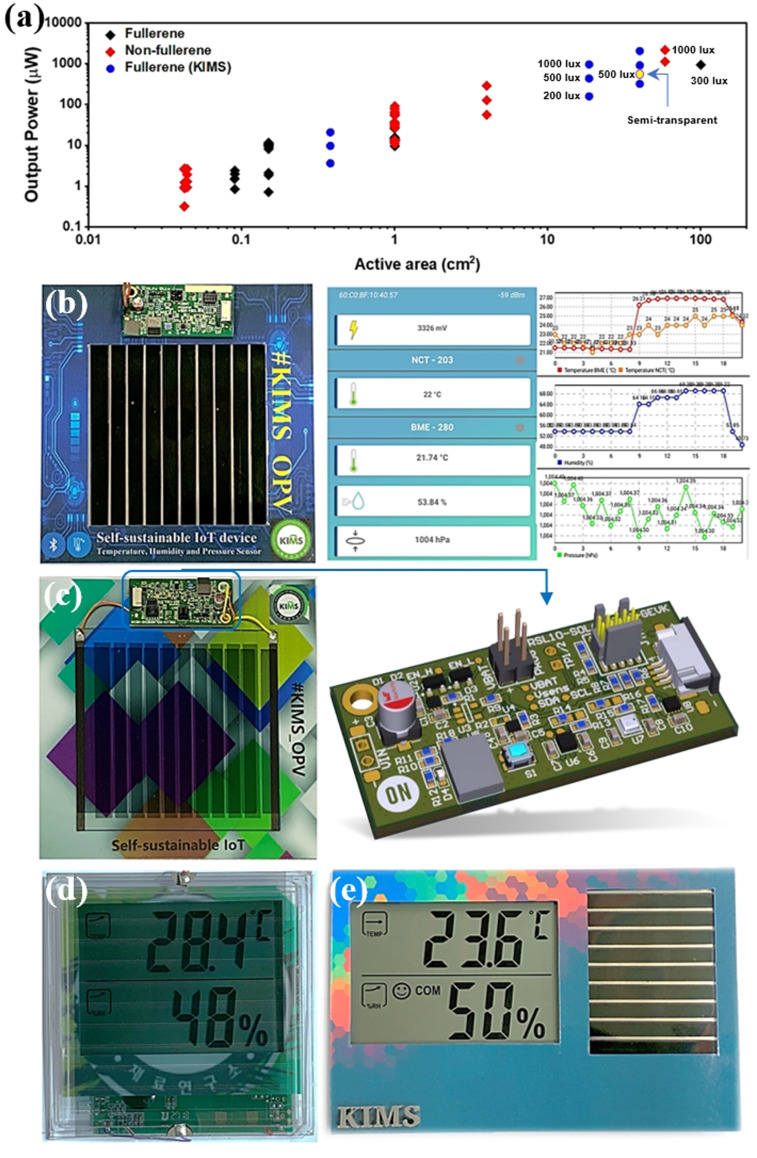
(a) Survey graph of indoor organic photovoltaics power generation with respect to active area. (b,c) Opaque and semitransparent OPV module integrated RSL‐10 multisensor IoT platform and android applications interface. (RSL‐10 schematic drawing source: ON Semiconductors). (d,e) Laboratory temperature and humidity meter powered by indoor semitransparent (10×10 cm^2^) and opaque (5×7 cm^2^) OPV modules.

Furthermore, owing to the very low energy flux of indoor lightening systems compare to standard outdoor irradiance (100 mW cm^−2^), indoor photovoltaics has not been seriously considered for practical applications. On the other hand, the lack of low power (<1 mW) electronic devices hindered the concept of self‐sustaining IoT systems. The development of low power sensors, microcontrollers and Bluetooth modules etc., opened new horizon for indoor photovoltaics integrated low consumption electronic applications. Our IOPV modules can provide output power of about 2 mW at 1000 lux illumination condition, which is quite sufficient to operate most of the low power Arduino based sensors and microcontrollers. A comparison of output power with respect to active area of recently reported IOPVs is shown in Figure 19a. One of the best features of the IoT device is to collect and communicate data over internet. For this purpose, we need to develop low power Wi−Fi modules whereas the commercially available Wi−Fi modules are still beyond the power limits of IOPVs with limited active area. One other option is the use of low power Bluetooth modules along with cell phone or computer software to store or communicate data over internet. Here, we use commercially available “RSL10‐SOLARSENS‐GEVK” multi‐sensor module manufactured by “ON Semiconductor” integrated with opaque and semitransparent IOPVs (Figure 19b,c). It contains temperature, humidity, pressure and axis sensors along with low power Bluetooth module and these sensors data can be collected on cellphone application (RSL10 Sensor Beacon). We integrated this sensor module with IOPV modules to develop a self‐sustaining IoT device for indoor application that work well under 500 lux illumination. Figure 19d,e exhibits the laboratory temperature and humidity meter that was connected with semitransparent IOPV (10×10 cm^2^) and 5×7 cm^2^ opaque mini‐module. Owing to very low power requirement (<50 μW), it can work well even with indirect light illumination direction under 300–500 lux. This work provides the basic prospect of OPV integrated IoT devices for indoor applications. Still a lot of efforts are required to design and develop efficient organic light absorber materials for indoor OPV applications along with the ultra‐low power IoT devices.

## 6 Summary and Outlook

In this Review, we have discussed the recent advances in IPV technologies, design rules, market trends, and future prospects for highly efficient IPVs. Prime focus has been given to the research and development of IOPVs, which have demonstrated great potential for IPVs, owing to their bandgap tunability, high absorbance coefficient, semitransparency, solution processability, and easy large‐area manufacturing through various printing techniques. The ultimate IOPVs can be realized by designing efficient donor and acceptor absorber materials with good spectral response in the visible region and better energy‐aligned interfacial layers, and through modulation of optical properties. The PCEs of IPVs are still lower than the theoretical values, with optimal energy bandgaps between 1.82 to 1.96 eV. Therefore, considering the theoretical PCE limits of IPVs, much effort is required to further improve the device performance of IOPVs.

There are a few fundamental factors that need to be considered carefully to attain excellent photovoltaic device performance under indoor light conditions. Firstly, the IPVs should have a good spectral response that matches the indoor lighting systems. That means that the absorbance spectra of IPV photoactive layers should overlap with the emission spectra of indoor light sources. Secondly, the EQEs of IPVs should be high to realize the maximum conversion of incident light photons into current, as well as to suppress the thermalization of photogenerated charges. Thirdly, the trap‐assisted charge recombination should be reduced, as low light intensities cause low carrier densities that can promote the trap assisted recombination mechanisms. Lastly, the energy losses of the IPVs should be low to realize the maximum *V*_OC_. As, reduced energy losses and high *V*_OC_ under low light intensities could boost the IPVs device performance. Although, large‐area OPVs are exclusively studied to optimize the large‐scale manufacturing process through different solvent systems and manufacturing techniques but there are only few reported about upscaling of OPVs for low‐light or indoor applications. In recent years, the development of high efficiency photo absorber materials and innovative fabrication techniques that are compatible with large scale, low‐cost manufacturing processes, demands that attention be focused on the operational stability of OPVs for commercialization. Numerous efforts have been devoted to understanding the degradation mechanism of OPVs under standard simulated illumination conditions or outdoor in direct sunlight, whereas there are only few reported studies into the degradation behavior of OPVs under indoor low‐light environments. However, relatively mild indoor conditions, compared to the severe outdoor or intense stability evaluation conditions such as heat, solar irradiation, and fluctuating weather, can provide a pathway for prolonged device operational stability of IOPVs.

IOPV device design is a challenging task, as the device optimizations carried out under standard illumination conditions could alter under low light illumination, leading to the poor device performance. Also, the different light illumination sources with nonuniform light intensities and spectral response could affect the device performance significantly. A comprehensive discussion of various approaches such as semitransparent IOPVs, anti‐reflection coatings, microstructures and surface plasmon effect to improve the device performance of IOPVs through light management that can reduce the effect of nonuniform and directional dependency of IOPVs under low light illumination conditions has been presented. Low power IoT devices integrated with photovoltaics have great prospects for health‐care, environmental monitoring, smart homes, smart markets and industry. The concept of IoT is beyond the limits where everything is connected for communication purposes with everything else. To meet the growing demands of product integrated photovoltaics for indoor applications, the researchers need to focus on the design and development of ultra‐low power IoT products and highly efficient IPVs. Although indoor IoT systems have the motivation of ultra‐low power consumptions, these applications are not limited to sensors such as motion, temperature, and humidity. The concept of IoT devices is beyond the limits of a closed space to an ecosystem that benefits the human life through the environment wherein smart services are provided to utilize every activity anywhere and at any time. To fulfil the requirements of such systems, the researchers need not only to design ultra‐low‐power IoT system but also provide better energy management through creating efficient indoor energy‐harvesting systems by utilizing IOPVs.

## Conflict of interest

The authors declare no conflict of interest.

## Biographical Information

*Muhammad Jahandar received his Ph.D. degree in Advanced Materials and Chemical Engineering from the University of Science and Technology, Korea in 2018. He is a senior researcher of Energy and Electronic Materials Center at the Korea Institute of Materials Science (KIMS), Korea. His current research focuses on device engineering of solution‐processed organic photovoltaics*.

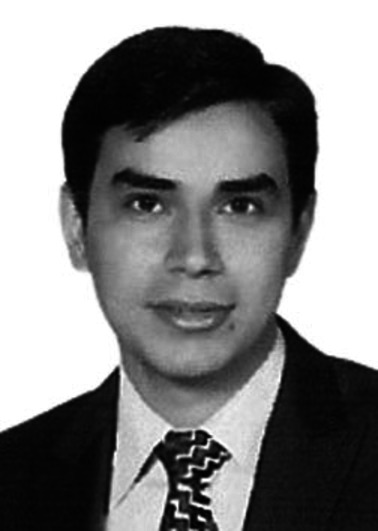

## Biographical Information

*Soyeon Kim received her Ph.D. degree in Chemical & Biomolecular Engineering from Yonsei University, Korea in 2017. She is a senior researcher of Energy and Electronic Materials Center at the Korea Institute of Materials Science (KIMS), Korea. Her main research interests are in organic materials including synthesis and applications for energy and electronic devices*.

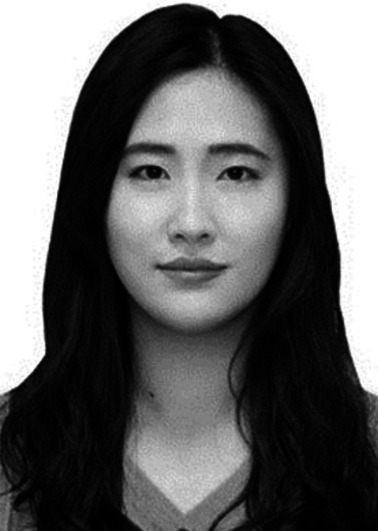

## Biographical Information

*Dong Chan Lim received his Ph.D. degree in Cluster Physics from Konstanz University, Germany in 2007. He is a principal researcher of the Energy and Electronic Materials Center at the Korea Institute of Materials Science (KIMS), Korea. His main research interests are in photovoltaics and related materials, such as quantum clusters and transparent electrodes. In addition, his research interests include photoelectrochemical devices and hybrid energy conversion materials to create clean and sustainable energy and water*.